# Greater Repertoire and Temporal Variability of Cross-Frequency Coupling (CFC) Modes in Resting-State Neuromagnetic Recordings among Children with Reading Difficulties

**DOI:** 10.3389/fnhum.2016.00163

**Published:** 2016-04-26

**Authors:** Stavros I. Dimitriadis, Nikolaos A. Laskaris, Panagiotis G. Simos, Jack M. Fletcher, Andrew C. Papanicolaou

**Affiliations:** ^1^Artificial Intelligence and Information Analysis Laboratory, Department of Informatics, Aristotle UniversityThessaloniki, Greece; ^2^Neuroinformatics Group, Department of Informatics, Aristotle UniversityThessaloniki, Greece; ^3^School of Medicine, University of CreteCrete, Greece; ^4^Department of Psychology, University of HoustonHouston, Texas, TX, USA; ^5^Division of Clinical Neurosciences, Department of Pediatrics, University of Tennessee Health Science CenterMemphis, TN, USA; ^6^Le Bonheur Neuroscience Institute, Le Bonheur Children's HospitalMemphis, TN, USA

**Keywords:** magnetoencephalography, dyslexia, resting state, phase-amplitude coupling, cross-frequency coupling, functional connectivity, chronnectome analysis, oscillopathies

## Abstract

Cross-frequency, phase-to-amplitude coupling (PAC) between neuronal oscillations at rest may serve as the substrate that supports information exchange between functionally specialized neuronal populations both within and between cortical regions. The study utilizes novel algorithms to identify prominent instantaneous modes of cross-frequency coupling and their temporal stability in resting state magnetoencephalography (MEG) data from 25 students experiencing severe reading difficulties (RD) and 27 age-matched non-impaired readers (NI). Phase coherence estimates were computed in order to identify the prominent mode of PAC interaction for each sensor, sensor pair, and pair of frequency bands (from δ to γ) at successive time windows of the continuous MEG record. The degree of variability in the characteristic frequency-pair PAC^f1−f2^ modes over time was also estimated. Results revealed a wider repertoire of prominent PAC interactions in RD as compared to NI students, suggesting an altered functional substrate for information exchange between neuronal assemblies in the former group. Moreover, RD students showed significant variability in PAC modes over time. This temporal instability of PAC values was particularly prominent: (a) within and between right hemisphere temporo-parietal and occipito-temporal sensors and, (b) between left hemisphere frontal, temporal, and occipito-temporal sensors and corresponding right hemisphere sites. Altered modes of neuronal population coupling may help account for extant data revealing reduced, task-related neurophysiological and hemodynamic activation in left hemisphere regions involved in the reading network in RD. Moreover, the spatial distribution of pronounced instability of cross-frequency coupling modes in this group may provide an explanation for previous reports suggesting the presence of inefficient compensatory mechanisms to support reading.

## Introduction

The neurophysiological basis and functional significance of spontaneous brain activity remains disputable among neuroscientists, ever since the initial demonstration of focally reduced activity associated with increased cognitive demands (Pinneo, 1966). More recently, clinical applications of spontaneous connectivity patterns have been explored in search of indices of abnormal brain function using a variety of imaging methods, including structural MRI, diffusion MRI, and functional MRI, electroencephalography (EEG) and magnetoencephalography (MEG; for reviews see Bassett and Bullmore, 2009; Sporns, 2014; Braun et al., 2015). Resting-state functional connectivity has been studied in relation to disorders of consciousness (Heine et al., 2012), Alzheimer's disease (Buckner et al., 2008; Stam et al., 2009; Khazaee et al., 2015), schizophrenia (Bassett et al., 2012; Damaraju et al., 2014), depression (Jin et al., 2011; for reviews see Rosazza and Minati, 2011; Dutta et al., 2014), in mTBI (Dimitriadis et al., 2015d; Antonakakis et al., 2016), in dyslexia (Koyama et al., 2010, 2011; Dimitriadis et al., 2013b), and in normal populations (Raichle et al., 2001; Greicius et al., 2003; Fair et al., 2008; De Pasquale et al., 2010; Brookes et al., 2011).

In this line of research, the brain is viewed as a complex network comprised of sets of smaller subsystems, interacting in a dynamic manner, both locally and at longer ranges (Tognoli and Kelso, 2014a,b). Local neuronal assemblies specialized for a particular neuronal process are tuned to generate oscillations in specific frequency bands, and that the interaction of a given assembly with more distant neuronal assemblies is “carried” by signals in the prominent oscillating frequency of the source population (Shew et al., 2009). This cross-frequency coupling (CFC) can support the encoding of nested hierarchical relations, which is crucial for the representation of composite objects, likely following specific syntactic rules known to both sender and receiver, and enables interpretation of long uninterrupted signals by the latter (Buzsáki, 2010). The same principle may apply to the communication between neuronal assemblies located within the same cortical region. This mechanism promotes accurate timing between different oscillatory rhythms and dynamic control of distributed functional networks (Varela et al., 2001; Buzsáki, 2006; Canolty and Knight, 2010). There is rapidly accumulating experimental evidence supporting this role of CFC in cognition (Jensen and Colgin, 2007; Canolty and Knight, 2010; Palva and Palva, 2011; Buzsáki and Watson, 2012; Jirsa and Müller, 2013; Dimitriadis et al., 2015a,c; Dimitriadis et al., 2016).

Findings pooled from several studies indicate that temporal parsing of neuronal activity in different frequency ranges is very well-conserved in mammalian evolution (Northcutt, 1995). This preservation suggests that temporal coordination of distributed brain processes, as reflected by oscillatory patterning, synchronization, phase locking, and cross-frequency coupling, might have important functions and not be epiphenomenal (Buzsáki et al., 2013). Such fine-tuned processes can be disrupted by a number of developmental variations including: (i) Changes in the subunit composition of ligand- and voltage-gated membrane channels altering the time constants and resonance properties of neurons and microcircuits; (ii) alterations in white matter integrity such as myelination and axonal diameter affecting conduction velocity and overall efficiency of neuronal signaling. In all of these variations one expects to find alterations in variables reflecting brain dynamics, such as the power of particular oscillations and the extent and precision of synchronization in the various frequency ranges and their cross-frequency relationships (Buzsáki and Watson, 2012).

In this context, appropriate descriptors of brain dynamics derived from EEG and MEG recordings may help characterize the pathophysiological substrate of psychiatric and developmental disorders in the form of “oscillopathies” or “dysrhythmias.” With respect to reading and associated developmental difficulties, there is accumulating evidence that the degree of myelination in left hemisphere cortico-cortical tracts correlates positively with reading skill (Niogi and McCandliss, 2006; Hoeft et al., 2011). In a recent fMRI study (Richards et al., 2015) reported strong associations between measures of white matter integrity assessed through Diffusion Tensor Imaging and functional connectivity using fMRI in several brain regions in children varying in reading and spelling ability. Interestingly, degree of connectivity was both down- and up-regulated among struggling readers in complex ways.

Distinct forms of cross-frequency interactions have been described: (i) power to power, (ii) phase to phase, (iii) phase to frequency and, (iv) phase to power (Jensen and Colgin, 2007). The latter type, also referred to as phase-amplitude modulation, has been repeatedly described in both animal and human data (Osipova et al., 2008; Cohen et al., 2009a,b; Colgin et al., 2009; Axmacher et al., 2010a,b; Tort et al., 2010) and is perhaps the most suitable CFC type to support high-precision timing of signaling between neuronal assemblies located either within the same or in remote cortical regions. This form of CFC can be estimated through the phase-to-amplitude coupling index (PAC) which quantifies the modulation of the amplitude of higher frequencies by the phase of slower rhythms. PAC captures the phenomenon whereby a specific number of cycles of (higher) frequency rhythms in the target population can be oscillated within the phase of slower rhythms of the source population. This number is presumed to reflect the amount of information that can be exchanged between the two neuronal oscillators operating at different characteristic frequencies. The PAC index quantifies both the strength of the interaction and the prominent frequencies displayed by each interacting pair of oscillators at each point in time (i.e., time window of recorded activity). In the present study PAC serves as an index of the neuronal communication mechanism whereby slower rhythms can reset ongoing neurophysiological processes taking place within a different neuronal population (Buzsáki, 2010; Canolty and Knight, 2010; Buzsáki et al., 2013). Further analyses on PAC data can be used to establish the repertoire of CFC modes that characterize resting activity in a particular group of participants (i.e., the different pairs of frequencies characterizing the modulating and the modulated neural oscillators across sensors). To date there is very little research on the typical patterns of CFC interactions in the normal mature brain, while even less is known regarding the repertoire of prominent CFC modes in typically developing children.

The amount of information being exchanged between two neuronal oscillators may increase as a function of the spectral separation between the two prominent oscillating frequencies (Jensen and Colgin, 2007; Canolty and Knight, 2010; Florin and Baillet, 2015). It is not known, however, whether the typical functional substrate for cortico-cortical signaling features a small number of CFC modes or if a larger repertoire of CFC modes is related to more efficient communication between neuronal assemblies. In this newly emerging field, establishing distinct profiles of CFC modes that vary systematically as a function of reading ability in children would provide important clues regarding the functional significance of PAC metrics.

In the case of neurophysiological activity recorded at rest, CFC patterns should be relatively stable in time if they are to serve as the substrate of functional interactions between specific neuronal assemblies. Although by definition neuronal communication is a dynamic process, only recently appropriate analytical tools were made available to model connectivity patterns as they evolve over time during the relatively restricted time window of a resting activity recording session. Analytic tools developed in the context of the theory of Coordination Dynamics (Tognoli and Kelso, 2014a,b) and used in the present study permit estimation of the probability of change from one prominent CFC mode to another over successive time windows, separately at each sensor and between sensor pairs, through the transition rate index (TR). Next, TR values are aggregated over time windows and sensors to provide global indices of the degree of CFC variability that characterizes resting state recordings of each participant. The derived indices can then be used to classify participants based on reading-group membership and further validated through associations with reading achievement measures.

This study had three aims: First, to identify prominent patterns of CFC interactions in typically developing school-aged children and to determine if similar modes characterize the resting state neuromagnetic recordings of an age-matched group of students who experience severe reading difficulties (RD); Second, to test the prediction that struggling readers would show more variable patterns of CFC over time than typical readers, suggesting a less stable substrate available to support the integration of signaling between neuronal assemblies. Finally, the potential functional significance of putative aberrant dynamic patterns of CFC for reading achievement was assessed through brain-behavior correlational analyses.

## Materials and methods

### Participants

The target group included 25 children (12 boys) with reading difficulties (RD group) as indicated by scores below the 16th percentile level (standard score of 85) on the Basic Reading composite index (average of Word Attack and Letter–Word Identification subtest scores of the Woodcock–Johnson Tests of Achievement-III; Woodcock et al., 2001; WJ-III). These scores would be consistent with the presence of dyslexia and is lower than in previous studies (Rezaie et al., 2011; Simos et al., 2011) of this cohort because we wanted to focus on severely impaired children. They were recruited from a larger Grade 6–8 educational study (Vaughn et al., 2010) at-risk for further academic failure because they did not pass the school- administered Texas Assessment of Knowledge and Skills (TAKS). The TAKS is a criterion-referenced reading comprehension assessment that represents the state accountability test. Additional standardized decoding assessments were administered to select this sample of participants because failure on the TAKS is not always because of decoding difficulties. Because entire middle schools were screened, this should ensure better representation of socioeconomic (SES), ethnic backgrounds, achievement, and general cognitive ability scores than recruiting through a clinic. While all of the struggling readers were invited and actively recruited to participate through letters to parents and follow-up phone calls, response rates for imaging studies in school—identified samples are typically very small (e.g., Simos et al., 2007; Meyler et al., 2008).

A second group of 27 children (9 boys) who had never experienced difficulties in reading (NI group) served as comparisons having standard scores >90 on the Reading Composite index (corresponding to the 36th percentile). These students were recruited from the same schools as the RD group in an attempt to control for educational history, ethnicity, and SES factors. All participants had Full Scale IQ scores >80 on the Wechsler Abbreviated Scale of Intelligence (Wechsler, 1999).

Detailed demographic and psychoeducational data are presented in Table 1 and graphically in Section 10 in Supplementary Material. The two groups were comparable on age, ethnicity, handedness, and Performance IQ (*p* > 0.1 in all cases). As expected the RD group scored significantly lower than the NI group on measures of reading, spelling, and Verbal IQ. Additionally, participants were selected for inclusion in either group only if they had T scores below 65 on the Attention and Hyperactivity CBCL scales (Child Behavior Checklist, Parental Form; Achenbach, 1991) or a mean score lower than 1.67 points on the parental ADHD-C SNAP-IV scale (Swanson et al., 2005), indicating low risk for ADHD (Chen et al., 1994). Informed assent and consent forms were signed by all participating children and their parents or guardians, respectively. The study had been approved by the University of Texas, Institutional Review Board.

**Table 1:** **Demographic and psychoeducational data for typical (NI) and students with severe reading difficulties (RD)**.

	**Group**	**Mean**	**SD**	**Range**
Age (years)	NI	11.35	2.8	7-14
	RD	12.20	2.1	7-14
LWID^**^	NI	99.55	8.9	90-126
	RD	80.73	8.2	62-85
WA^**^	NI	99.44	9.6	91-130
	RD	84.78	7.2	68-85
Composite^**^	NI	99.70	9.7	90-130
	RD	81.78	6.9	65-85
VIQ^*^	NI	104.04	16.6	80-141
	RD	90.76	13.3	81-128
PIQ	NI	100.29	11.2	80-117
	RD	95.39	12.6	80-129

Group differences:

* *p < 0.01*,

** *p < 0.001*.

*Abbreviations, LWID, Woodcock-Johnson III Letter-Word Identification; WA, Woodcock-Johnson III Word Attack; Composite, Woodcock-Johnson III Basic Reading composite; VIQ, WASI Verbal IQ score; PIQ, WASI Performance IQ score*.

### MEG recordings

Three minutes of continuous brain activity was acquired with a whole-head neuromagnetometer array (4-D Neuroimaging Magnes WH3600), consisting of 248 first-order axial gradiometer coils, housed in a magnetically shielded chamber. Participants were placed in a supine position and instructed to keep their eyes closed during the recording. Data were originally collected at a sampling rate of 1017.25 Hz and bandpass filtered within 0.1–200 Hz. Axial gradiometer recordings were then transformed to planar gradiometer field approximations using the *sincos* method available in Fieldtrip (Oostenveld et al., 2011).

### Data preprocessing

Ongoing activity was corrected for artifacts through a two-step procedure implemented in Matlab (The MathWorks, Inc., Natick, MA, USA) and Fieldtrip (Oostenveld et al., 2011). Line noise was first removed using a notch filter at 60 Hz and the single-subject data was whitened and reduced in dimensionality by means of Principal Component Analysis (PCA) with a threshold corresponding to 95% of total variance (Delorme and Makeig, 2004; Escudero et al., 2011; Antonakakis et al., 2013). The resulting signals were submitted to independent components analysis (ICA) using the extended Infomax algorithm as implemented in EEGLAB (Delorme and Makeig, 2004). A given independent component was considered to reflect ocular or cardiac artifacts if more than 30% of its z-score kurtosis or skewness values, respectively, were outside ± 2 of the distribution mean (Escudero et al., 2011; Antonakakis et al., 2013). The remaining ICs were used to reconstruct a relatively artifact-free signal. The average number of artifactual ICs was 5.5 for NI and 5.1 for RD students. To minimize the contribution of volume conduction to the estimates of intrinsic coupling modes (Engel et al., 2013) and to the identification of the dominant cross-frequency pairs, MEG recordings were orthogonalized prior to dynamic cross-frequency coupling analysis (Hipp et al., 2012). Next, the data were bandpass-filtered in the following frequency ranges using a Third-order Butterworth filter (in zero-phase mode): 0.5–4, 4–8, 8–10, 10–13, 13–15, 15–19, 20–29, and 30–45 Hz corresponding to δ, θ, α_1_, α_2,_ β_1_, β_2_, β_3_, and γ bands.

### CFC metric computation

CFC estimates the strength of pairwise interactions and identifies the prominent interacting pair of frequencies, both between and within sensors (Buzsáki, 2010; Canolty and Knight, 2010; Buzsáki et al., 2013). Among available CFC descriptors, phase-amplitude coupling (PAC), which relies on phase coherence, is the one most commonly encountered in research (Cohen et al., 2009a,b; Voytek et al., 2010). The PAC algorithm, as adapted to continuous MEG multichannel recordings, is described below.

The more general case of CFC (i.e., between-sensor coupling) is described here—within-sensor CFC is derived by collapsing the two sensor indices to a common index. Let x(i_sensor_, t) be the MEG activity recorder at the i_sensor_-th site, and *t* = 1, 2,.…T the successive time points. Given two frequency-limited signals x(i_sensor_, t) and x(j_sensor_, t), cross-frequency coupling is estimated by allowing the phase of the lower frequency (LF) oscillation to modulate the amplitude of the higher frequency (HF) oscillation. The complex analytic representations of each signal z_LF_(t) and z_HF_(t) are derived via the Hilbert transform (HT[.]).

$$\begin{array}{l} {\text{z}}_{\text{LF}}(\text{t})=\text{HT}[{\text{x}}_{\text{LF}}(\text{t})]=\left|{\text{z}}_{\text{LF}}(\text{t})\right|\;{\text{e}}^{\text{i }\phi_{\text{LF}}(t)}=A_{\text{LF}}(\text{t})\;{\text{e}}^{\text{i }\phi_{\text{LF}}(t)},\;\\ {\text{z}}_{\text{HF}}(\text{t})=\text{HT}[{\text{x}}_{\text{HF}}(\text{t})]=\left|{\text{z}}_{\text{HF}}(\text{t})\right|\;{\text{e}}^{\text{i }\phi_{\text{HF}}(t)}=A_{\text{HF}}(\text{t})\;{\text{e}}^{\text{i }\phi_{\text{HF}}(t)} \end{array}$$

Next, the envelope of the higher-frequency oscillation A_*HF*_(t) is bandpass-filtered within the range of the LF oscillation and the resulting signal is submitted to an additional Hilbert transform to derive its phase dynamics component ϕ'(t):

$$\begin{array}{l} \text{z}\prime (\text{t})=\text{HT}[\;A_{\text{HF},\text{LF}}(\text{t})\;]=\;\left|\text{z}\prime (\text{t})\right|\;{\text{e}}^{\text{i }\phi \prime_{\text{HF}}(t)}=\left|\text{z}\prime (\text{t})\right|\;{\text{e}}^{\text{i }\phi_{\text{LF}\to \text{HF}}(t)} \end{array}$$

which expresses the modulation of the amplitude of the HF by the phase of the LF oscillation. Phase consistency between the two time series is measured by means of both the original definition (Lachaux et al., 1999) and the imaginary portion of PLV (iPLV), as synchronization indexes to quantify the strength of PAC.

The original *PLV* is defined as follows:

(1) $$\begin{array}{l} PLV=\frac{1}{T}*\left|\sum_{t=1}^{T}e^{i(\phi_{LF}(t)-\phi_{HF}(t))}\right| \end{array}$$

and the imaginary part of *PLV* as follows:

(2) $$\text{Im}PLV=\frac{1}{T}*\left|Im\left(\sum_{t=1}^{T}e^{i(\phi_{LF}(t)\;-\;\phi_{HF}(t))}\right)\right|$$

The imaginary portion of PLV is considered to be less susceptible to volume conduction effects in assessing CFC interactions and was used in all subsequent analyses. While iPLV is not affected by volume conduction, it could be sensitive to changes in the angle between two signals, which do not necessarily imply a PLV change. In general, iPLV is only sensitive to non-zero-phase lags and is thus resistant to instantaneous self-interactions associated with volume conductance (Nolte et al., 2004). For comparison purposes results based on PLV are shown as Supplementary Material (Section 7).

**Figure 1:**
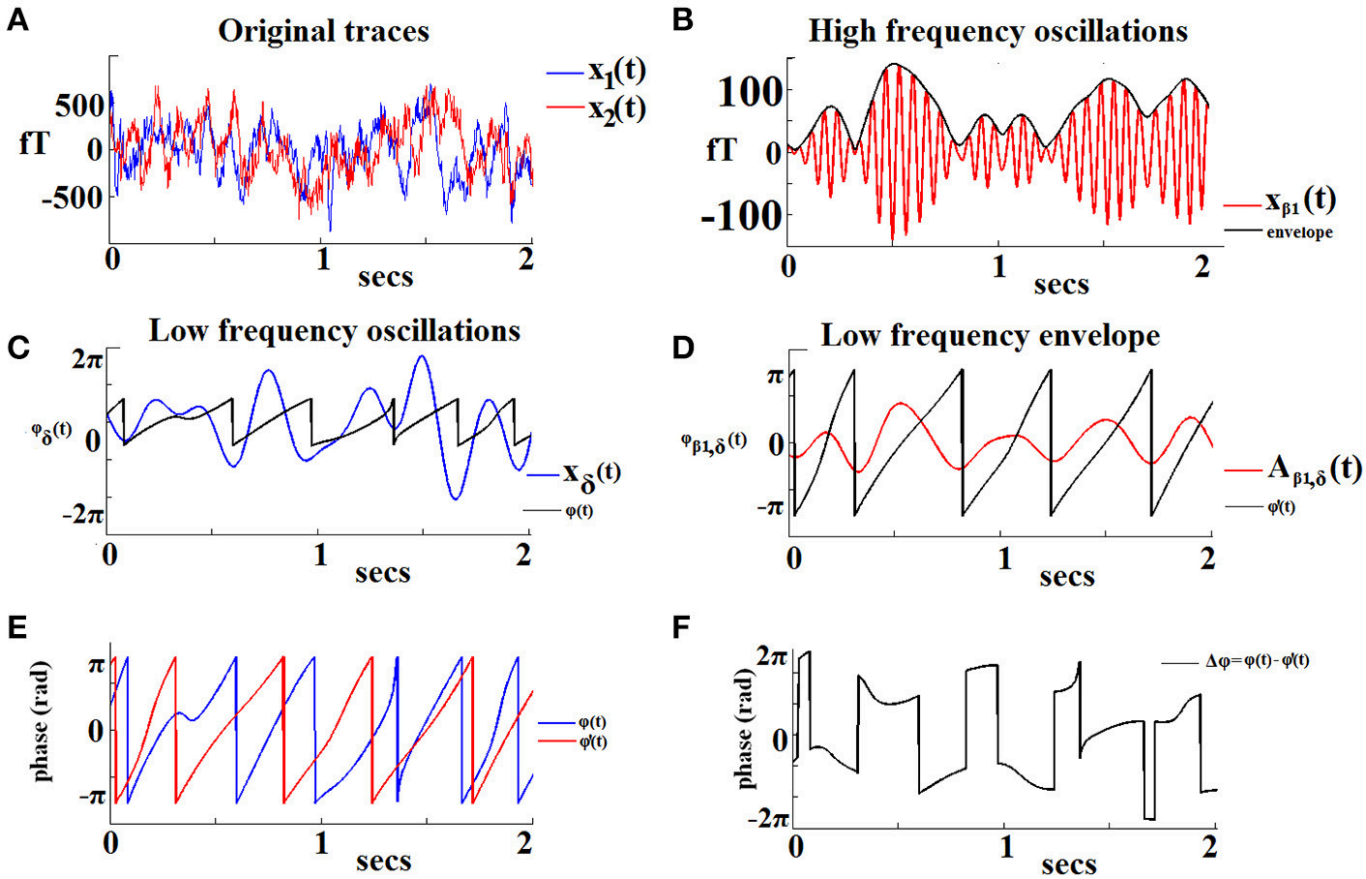
**An example of the algorithmic steps involved in the estimation of iPLV between the phase of δ oscillations recorded from a left frontal sensor (LF signal) and the amplitude of β1 oscillations recorded from a right frontal sensor (HF signal)**. The two continuous MEG time series are shown in **(A)**. The HF signal filtered in the β_1_-band is shown in (**B**; red trace) with its amplitude modulation envelope superimposed (dark trace). Panel **(C)** displays the LF signal filtered in the δ-band (blue trace) along with its corresponding instantaneous phases φ'δ(t) (dark trace). The δ oscillations in the HF signal are shown in (**D**; red trace) with the corresponding instantaneous phases superimposed [φ'δ(t)]. The evolution of instantaneous phases of the LF rhythm overlaid on the corresponding phases of the HF oscillation envelope is shown in **(E)**. The evolution of phase differences between the two signals (Δφ) is used as an index of frequency-specific phase-amplitude coupling (PAC) between the two sensors **(F)**.

Figure 1 demonstrates the aforementioned algorithmic steps for a pair of sensors located over left and right frontal areas in a specific 2-s sliding window from the recording in a NI participant. In this example we show PAC interactions between oscillations in the δ band (LF) and oscillations in the β_1_ band (HF). The original recordings are shown in Figure 1A. The HF component of signal x1(t) with its envelope is depicted in Figures 1B,D shows the low-pass filtered (within the δ band) version of the previous envelope [signal denoted A_β__1, θ_(t)]. The saw-like trace corresponds to its instantaneous phases $\phi_{\beta }^{\prime }$_1_(t). The LF version of the signal x2(t) is depicted in Figure 1C, along with the trace of the corresponding instantaneous phases ϕ_θ_(t). The ϕ_θ_(t) and $\phi_{\beta }^{\prime }$_1_(t) traces are superimposed in Figure 1E, to reveal the instantaneous phase differences which are plotted in Figure 1F. It is this sequence of phase-differences Δϕ(t) that enters in Equation (1) and will be “integrated” across time via averaging the corresponding directional vectors e^*i*Δ*ϕ*(*t*)^ in the complex domain. It is important that the length T of this sequence is sufficiently long so that the derived iPLV index provides a reliable estimate of PAC.

For each pair among the 248 MEG sensors, at each sliding window and for each subject, the maximum PAC score was computed for each combination of *f*φ (frequency band of the phase modulators from δ to γ) and *fa* (frequency modulated oscillator ranging from 0.5 to 45 Hz in 1 Hz steps. The maximum PAC value for each pair of sensors and at each sliding window in both the amplitude and phase domain is given by the following equation:

(3) $$\begin{array}{l} {(}^{\wedge }{\text{f}}_{\phi }{,}^{\wedge }f_{\alpha })=\underset{{\text{f}}_{\phi }\text{-}f_{\alpha }}{\arg\max}(PAC({\text{f}}_{\phi },\;f_{\alpha })) \end{array}$$

In this manner we were able to assess if the prominent CFC interaction (indexed by the maximum PAC value), and specifically the frequency of the low-frequency phase, was associated with the frequency of maximum power. If the two frequencies were identical this would imply that the observed prominent CFC interaction was driven by the power of the dominant frequency and not its phase. In order to ensure that we did not include in further analyses CFC interactions of this type, we required a minimum frequency difference of 1 Hz distance between the two frequencies (frequency of the low-frequency phase, frequency of the higher power), the one identified by Equation 3 (maximum PAC value) and the one associated with the maximum spectral power, within the range of high amplitude. Section 1 in Supplementary Material describes the method employed to assess whether PAC estimates were not biased by the maximum power in more detail.

#### Dynamic PAC estimates: the time-varying CFC graph (^TV^PAC-graph)

The goal of the analytic procedures described in this section was to capture the repertoire of CFC interactions and their temporal evolution, while taking into account the quasi-instantaneous spatio-temporal distribution of PAC estimates. This was achieved by computing a set of PAC estimates within each series of sliding, partially overlapping windows spanning the entire 3-min continuous MEG recording. The width of the temporal window was set equal to the duration of two cycles of δ activity (i.e., 2 s) ensuring that modulations of activity by the phase of the lowest frequency band (δ) would be preserved. The center of the sliding window moved forward at 0.5-s steps and the CFC interactions between every possible pair of frequencies were reestimated leading to a temporally integrated PAC graph. In this manner a series of 308 sets of PAC estimates were computed per sensor pair for each participant.

This procedure, the implementation details of which can be found elsewhere (Dimitriadis et al., 2010a), resulted in 217 time-varying PAC graphs per participant (^TV^PAC), each serving as an instantaneous snapshot of the surface network. There were 41, 37, 35, 32, 30, 26, and 16 (= 217) possible pairs in the δ, θ, α1, α2, β1, β2, and β3 bands. For instance, using 1 Hz frequency bins there were 45–4 = 41 interacting pairs for δ phase. ^TV^PAC tabulates PAC estimates between and within sensors.

#### Surrogate data analysis of PAC estimates

To identify significant PAC-interactions which were estimated for every pair of frequencies, within and between all 248 sensors, and at each successive sliding window, we employed surrogate data (Theiler et al., 1992). Surrogate data analyses determined: (a) if a given PAC value differed from what would be expected by chance alone, and (b) if a given non-zero PAC indicated coupling that was, at least statistically, non-spurious.

For every time window, sensor-pair, and pair of frequencies, we tested the null hypothesis H_0_ that the observed PAC value came from the same distribution as the distribution of surrogate PAC-values. One thousand surrogate time-series $\phi_{LF}^{s}\left(t\right)$ were generated by cutting at single point at a random location and exchanging the two resulting time courses (Canolty et al., 2006; Aru et al., 2015). Repeating this procedure produced a set of surrogates with minimal distortion of the original phase dynamics and impact on the non-stationarity of brain activity as compared to either merely shuffling the time series or cutting and rebuilding the time series in more than one time points. This procedure ensures that the observed and surrogate indices shared the same statistical properties. For each data set the surrogate PAC (^s^PAC) was computed. We then determined a one-sided *p*-value expressing the likelihood that the observed PAC value could belong to the surrogate distribution, and corresponded to the proportion of “surrogate”' PAC^s^ which was higher than the observed PAC value (Theiler et al., 1992). PAC values associated with statistically significant *p*-values were considered unlikely to reflect signals not entailing PAC coupling.

The FDR method (Benjamini and Hochberg, 1995) was employed to control for multiple comparisons (across all possible pairs of frequencies) with the expected proportion of false positives set to *q* ≤ 0.01. Finally, the PAC mode that characterized a specific pair of frequencies was determined based on the highest, statistically significant PAC value from surrogates. To be included in further analyses a given PAC value should meet an additional criterion, namely that the frequency of the modulated HF should be distinct from the frequency associated with maximum power (see Section 2.4. and Section 1 in Supplementary Material). Here, we adopted a criterion of at least 1 Hz difference between the two frequencies.

**Figure 2:**
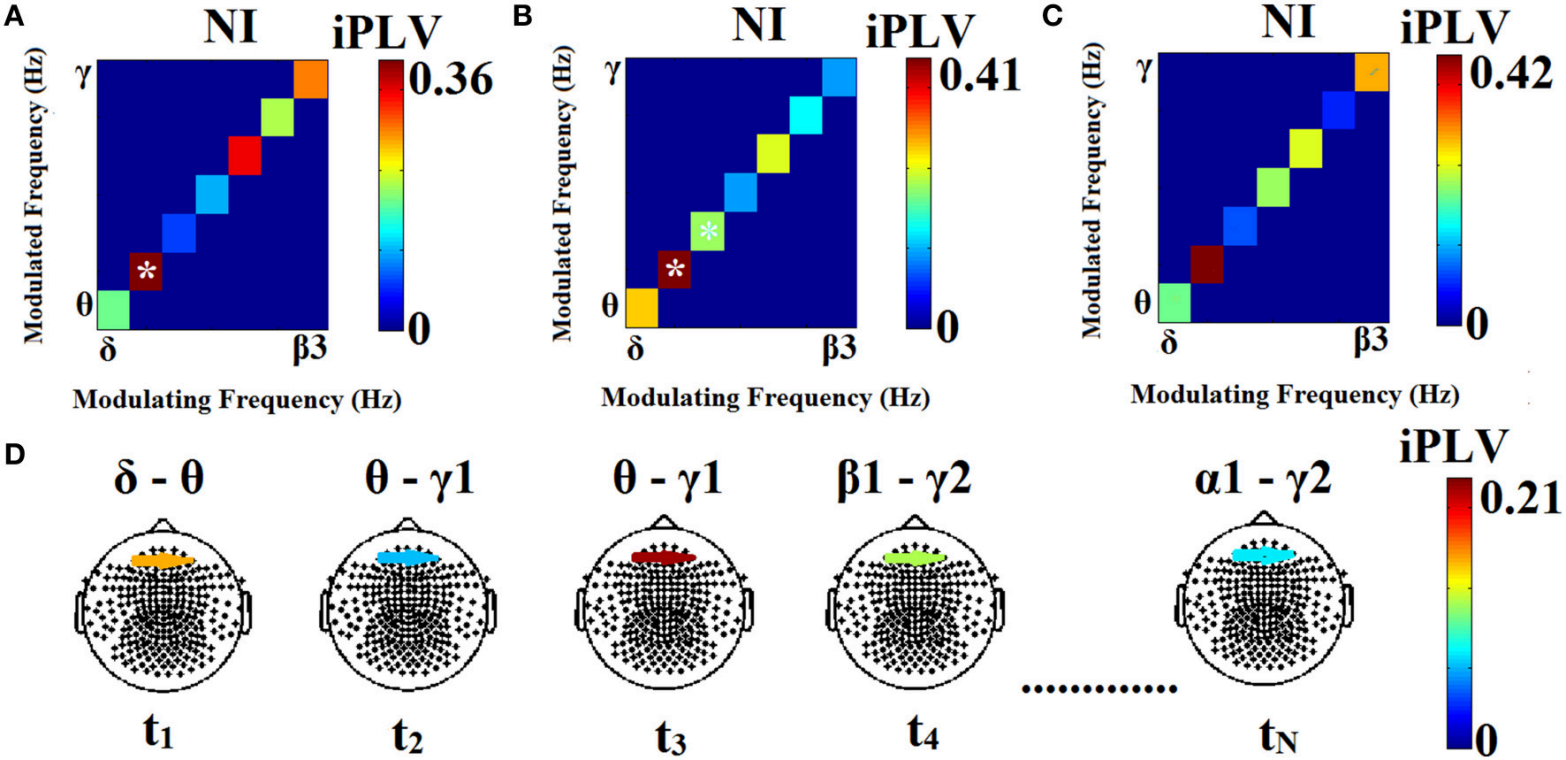
**Three different scenarios in the process of identifying prominent PAC modes associated with a particular pair of sensors at three different time windows (A–C)**. The modulating phase (LF) is plotted on the horizontal axis. iPLV values for each frequency pair are shown on the diagonal and are marked by (^*^) if they exceed the statistical threshold. A significant θ–α_1_ PAC value is shown in **(A)**, two significant frequency pairs (θ–α_1_ and α_1_–α_2_) were found in **(B)** with the former identified as the prominent pair, whereas PAC values failed to reach significance during the third window **(C)**. The sensor-frame location where data originated from is marked in **(D)**. The modulation of iPLV values across the entire MEG time series (successive time windows t1 …tN) is also shown in **(D)**. At t1 the phase of the LF (δ) of the signal recorded at a left frontal sensor appears to modulate θ amplitude at a right frontal sensor. At t2, there is modulation of right frontal γ1 amplitude by the phase of left frontal θ oscillations. At t3 this PAC mode persists, although its strength is higher. Subsequently (t4) the prominent PAC mode involves modulation of γ2 amplitude by β1 phase. For the two sensors shown here, the last time window (tN) is characterized by modulation of γ2 amplitude by the phase of α1 oscillations.

Finally, the significant, dominant PAC values for each pair of sensors and across sliding windows were integrated within each frequency band yielding 28 possible pairwise PAC estimates among the eight frequency bands. In the example of Figure 2A only one frequency pair met this criterion. In the case of two frequency pairs both exceeding the statistical threshold (Figure 2B), the one with the highest PAC value was identified as the characteristic PAC mode for this pair of sensors at that particular time window. If none of the cross-frequency pairs exceed the statistical threshold (Figure 2C), a value of zero was assigned to this pair of sensors with not identified characteristic coupling mode. For each participant the resulting ^TV^PAC profiles constituted a 4D array of size [28 (pairs of frequencies) × 308 (time windows) × 248 (sensors) × 248 (sensors)]. The identity of prominent frequency pairs for every pair of sensors (including within-sensor interactions) at each time window was finally stored in a second 4D array of size [28 × 308 × 248 × 248]. In the latter array significant PAC interactions were indicated by a value of 1, with zeros indicating non-significant PAC interactions.

### Group comparisons on CFC metrics

#### Dynamic strength of ^TV^PAC

Participants in the RD and NI groups were initially compared on the time-series of grand total PAC strength aggregated over all pairs of sensors. The dynamic strength of ^TV^PAC was estimated by aggregating the dominant cross-frequency pair and its strength estimated via iPLV over all pairs of sensors for each sliding window. We adopted two complementary indices, namely Dynamic Information Exchange Rate (dIER) and Dynamic Weighted Information Exchange Rate (wdIER), to aggregate both the dominant CFC mode and the strength over all pairs of sensors. Each approach resulted in a single time series per participant describing the temporal evolution of information exchange rate.

#### Repertoire of characteristic cross-frequency interactions

The two groups were further compared on the repertoire of prominent PAC modes which were derived with the procedure described in section 2.4.2. In this step, in order to reduce the number of comparisons, a single 7 × 7 matrix was obtained per participant by aggregating over all successive time windows and sensor pairs. The resulting empirical probability distributions (PDs) of all interacting pairs of frequencies were then averaged across participants to identify the prominent cross-frequency phase-amplitude coupling pairs for each group. Additionally, we constructed separate comodulograms (7 × 7 matrices) aggregated over time *or* sensor space (see Figure S2 in Supplementary Materials).

#### Transition dynamics based on dynamic prominent CFC profiles

Next, ^TV^PAC measurements were used in order to assess group differences in the *stability* of PAC modes. An index of the dynamic characteristics of CFC was computed in the form of the transition rate (TR) defined as the number of times that a given PAC profile at latency t (e.g., θ → β1) changed during the next latency into a different characteristic interaction (e.g., θ → γ), divided by the total number of possible transitions. TR can take values between 0 and 1 and is defined as follows:

(4) $$\begin{array}{r} TR=\frac{number\;of\;transitions\;between\;CFC\;pairs}{number\;of\;temporal\;segments-1} \end{array}$$

Using this formula a single TR value was computed for each participant, sensor (within-sensor CFC) and sensor pairs (between-sensor CFC). An example of cross-frequency pairs fluctuating over time is presented in Figure 2D, where the TR for the first four time windows would be TR = 2/(4–1) = 2/3 = 0.66. Transitions were counted only between two latencies where a significant CFC was detected.

#### Dynamic information exchange rate (dIER)

Under the notion that PAC modes reflect processes through which “packets of neural information” are exchanged between populations of neurons operating at different characteristic frequencies, we developed two novel measures to summarize the rate of information transfer between neural assemblies throughout the brain. These information theory estimates were based on the dominant PAC mode for each pair of sensors over time. The “instantaneous” (dynamic) Information Exchange Rate (dIER) is the sum of the number of cycles of the higher frequency that can be nested within the phase of the slower frequency across prominent frequency pairs and sensor pairs at each time window. The dIER index is defined as follows:

(5) $$\begin{array}{r} dIER\left(t\right)=\overset{No\;of\;sensors}{\underset{i_{sensor}=1}{\sum }}\overset{No\;of\;sensors}{\underset{j_{sensor}=1}{\sum }}\frac{No\;of\;cycles\;of\;HF}{No\;of\;cycles\;of\;LF} \end{array}$$

A weighted version of dIER (wdIER) was also computed that takes into account the strength of dominant PAC modes as indexed by the corresponding iPLV, with higher values implying stronger modulation of higher frequency rhythms by lower frequency oscillations throughout the recording epoch. Thus, in addition to providing information regarding how many cycles of the higher frequency can be “travelled” within the cycle of the lower frequency, wdIER also indexes how active is a “channel” between or within sensors for information exchange at quasi-stable time instances, and is defined as follows:

(6) $$\begin{array}{r} wdIER\left(t\right)=\overset{No\;of\;sensors}{\underset{i_{sensor}=1}{\sum }}\overset{No\;of\;sensors}{\underset{j_{sensor}=1}{\sum }}\frac{No\;of\;cycles\;of\;HF}{No\;of\;cycles\;of\;LF}*iPLV \end{array}$$

In the example of Figure 1, there were 2^*^14 cycles of β_1_ oscillations (HF) and 2 cycles for δ (LF) within the window of 2 s with a corresponding IER = 28/2 = 14. With a iPLV value of 0.21, wdIER = IER^*^iPLV = 14^*^0.21 = 2.94. The frequency bin (1 Hz resolution) of the faster oscillation where the maximum PAC was detected was used to compute this non-integer ratio of the two frequencies. A cycle of recorded activity in a particular frequency range corresponds to the period of the corresponding sinusoidal oscillation (see section 11 in supp. material).

#### Classification of RD and NI readers based on dIER/wdIER, TR profiles and PD^*PAC*^ of prominent CFC

Four complementary features were validated for their capacity to discriminate between NI and RD groups using a k-Nearest Neighbor (k-NN) classifier: the dynamic information exchange rate (dIER and wdIER indices) for each participant, the TR profiles, and the probability distribution (PD^PAC^) of prominent CFC (see **Figure 4**). The corresponding feature-vectors (FVs) input to the k-NN classifier consisted of: the dIER and wdIER time series (each consisting of 308 samples corresponding to the number of time windows), of a 248 × 248 matrix of TR values, and of a vector consisting of 28 PD^PAC^ values.

Classification performance was assessed in four adjunct validation schemes. The first scheme entailed *leave-one-out cross-validation* where each participant was considered of unknown group membership. On each run, the k-NN classifier was trained on data from a subset of 51 participants (27 NI + 25 RD –1) and tested on its capacity to correctly classify the remaining participant based on each FV separately (dIER, wdIER, TR, or PD^PAC^).

The second scheme was a *2-fold cross-validation*, where the k-NN classifier was first trained on data from a randomly created training data set from 26 participants with known group membership and tested for classification accuracy on the data from the remaining 26 participants. Again classification accuracy was tested separately for each of the four FVs.

The third and fourth validation schemes were conducted in order to evaluate the within-subject reliability of the four sets of features (dIER or wdIERs, TR and PD^PAC^) for discriminating RD from NI students. Initially, the dIER and wdIER time series were split into two segments of equal length and TR and PD^PAC^ values were recalculated for each segment. FVs input to the k-NN classifier consisted of either the 154D split-segment dIER and wdIER time series, the 248D TR value matrices, or the 28 PD^PAC^ values from each time segment and participant. The third validation scheme employed a *leave-one-out procedure* where indices derived from 51 × 2 segments served as the training set and the two segments from the remaining participant as the testing set. In the *2-fold cross-validation*, training and testing sets included data from 52 randomly mixed A and B segments from each participant with the remaining 52 segments serving as the testing set.

To properly account for the dynamics of information exchange rate represented by the dIER and wdIER time series, each 308D set (or 154D set in the split-temporal domain schemes) was first transformed into a dynamic trajectory using a time-delay embedding procedure and used to compute the Wald-Wolfowitz (WW) Dissimilarity Index (w; described in more detail in Section 8 of the Supplementary Material).

Results for each classification scheme were pooled over 200 repetitions employing different training and testing sets each time. The Euclidean distance served as the optimization criterion in classification runs based on TR and PD^PAC^ profiles, whereas the Dissimilarity Index was employed for classification runs conducted on the dIER and wdIER data sets, as a more appropriate distance estimator between time series (Friedman and Rafsky, 1981; Rigas et al., 2015; see section 8 in Supplementary Material). Classification results were robust for k parameter values ranging between 7 and 11.

#### Predictability of dynamic information flow

To access the predictability (or randomness) of dynamic information flow in both groups, we also estimated sample entropy (Richman and Moorman, 2000). Sample entropy was estimated over both dIER and wdIER indices in both groups while the pair of embedding dimensions and time delays were optimized for each time series accordingly (see Sections 8 and 9 in Supplementary Material).

## Results

### Dynamic strength of ^TV^PAC

To ensure that PAC values were not driven by the power of the low-frequency signal, we estimated the difference between: (a) the low frequency involved in computing each PAC value and (b) the frequency associated with the highest power in the signal, separately for each time window, sensor, and sensor pair (see Section 1, Supplementary Material). In more than 90% of all instances across subjects, the two frequencies differed by at least 1 Hz and in more than 70% of instances by at least 2 Hz. This implies that the frequency of the slower oscillator rarely corresponded to the dominant frequency in the power spectrum, supporting the claim that PAC reflects a phase-modulated phenomenon and is not driven by the amplitude of the dominant frequency.

**Figure 3:**
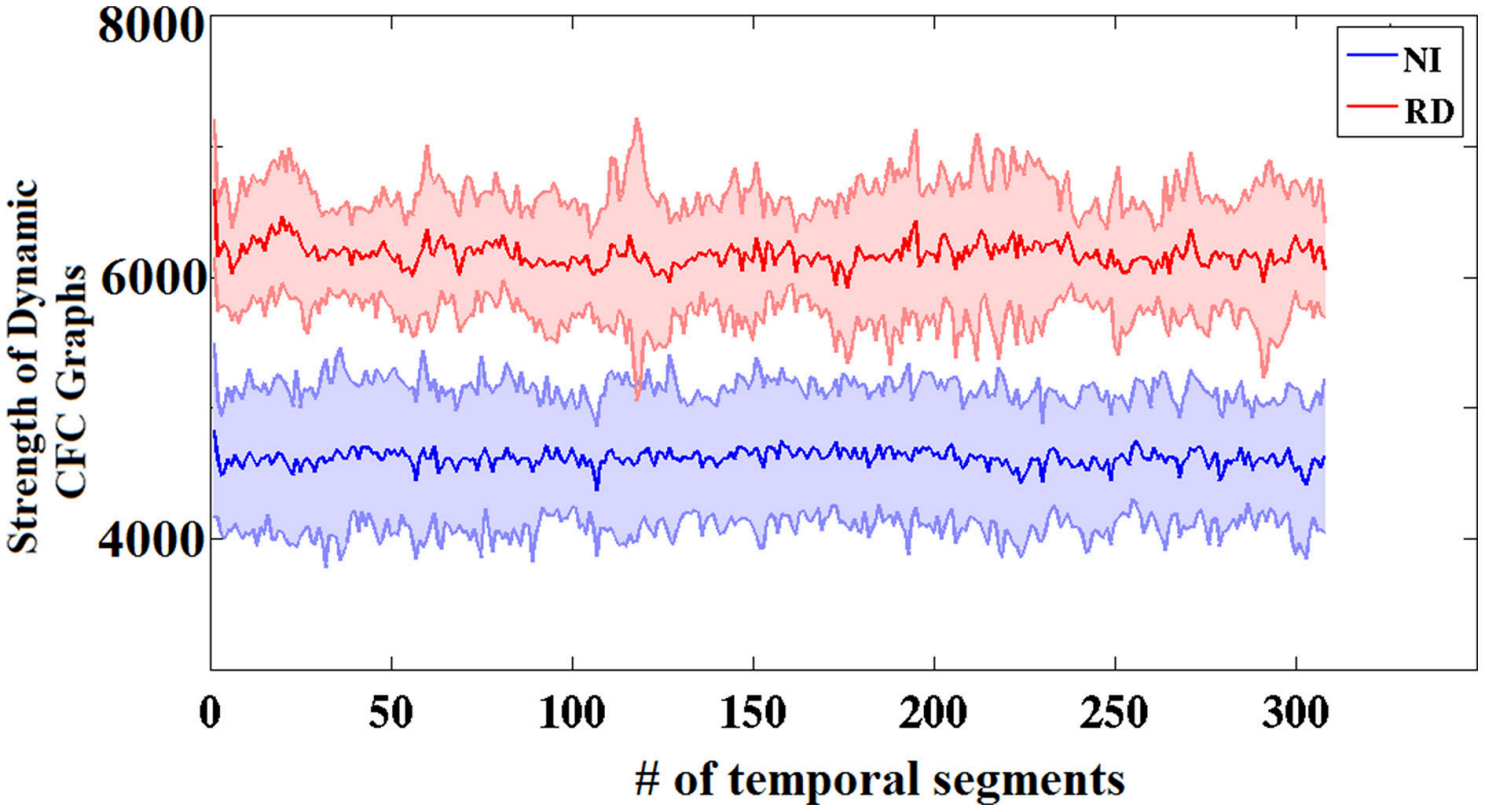
**Group-averaged strength of dynamic CFC graphs showing significantly greater overall strength in the RD compared to the NI group (Wilcoxon score, *p* < 10^−13^)**. The sums of PAC values over the entire network (between every possible pair of sensors) are plotted as solid lines surrounded by the corresponding ± 1 SD ranges.

Figure 3 presents the group-averaged traces of dynamic ^*TV*^PAC strength showing higher PAC levels for RD children. It is important to mention here that, since we are dealing with resting state data, the underlying brain activity is not aligned across subjects. Although group averaging can only be used for demonstration purposes, the distributions of individual ^*TV*^PAC values were clearly different between groups (Wilxocon test, *p* < 10^−12^).

### The repertoire of characteristic CFC interactions

**Figure 4:**
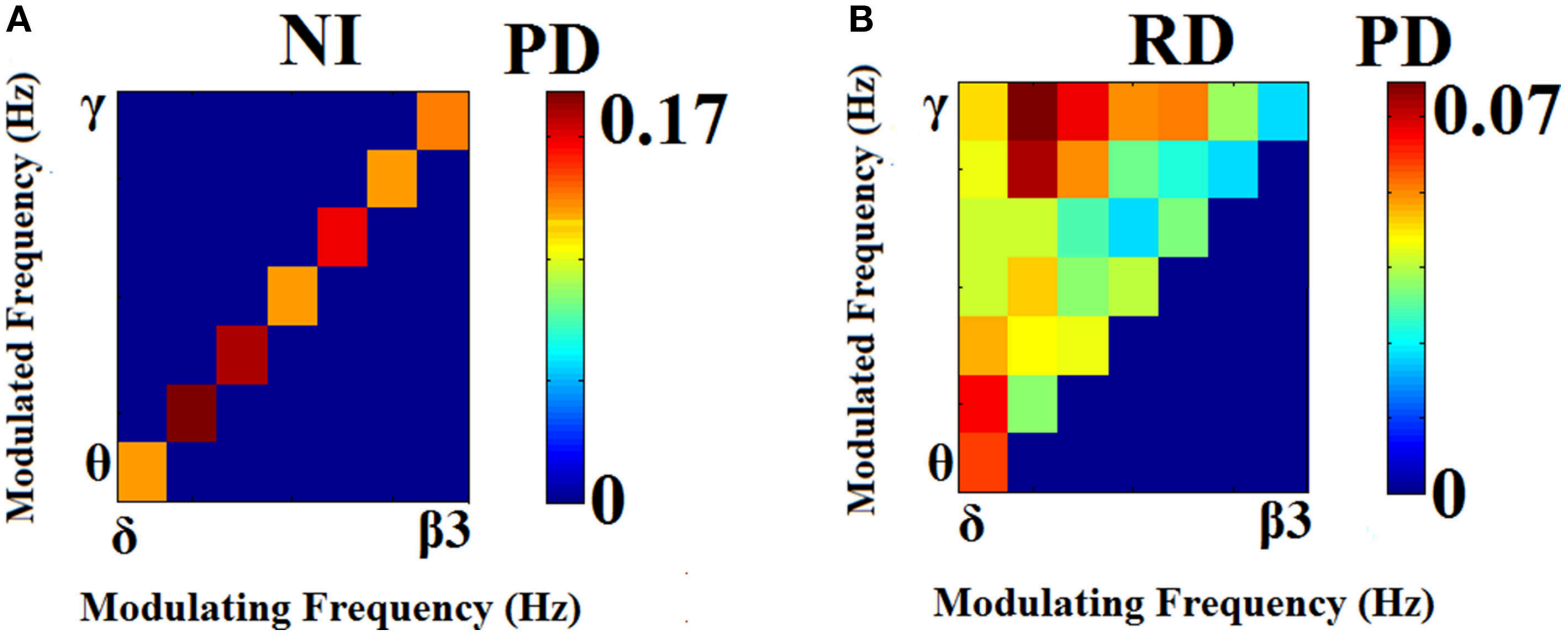
**Group-averaged empirical Probability Distribution values of cross-frequency phase-amplitude coupling pairs, revealing a broader repertoire of CFC pairs for RD (B) compared to NI (A) participants who showed a more restricted range of prominent CFC pairs**. Lower PD values indicate more significant iPLV values aggregated over time windows and sensor pairs.

Prominent frequency pairs (i.e., PAC modes) across all sensors, sensor pairs, and time windows were summarized for each subject in the form of an 8 x 8 matrix. Group-averaged for demonstration purposes, these empirical probability distributions presented in Figure 4 reveal a richer repertoire of CFC-interactions among RD children. In contrast, CFC interactions typical of NI children occur between brain rhythms in spectrally adjacent frequency bands (e.g., δ → θ, θ → α1 etc).

### Transition dynamics of prominent CFC interactions

**Figure 5:**
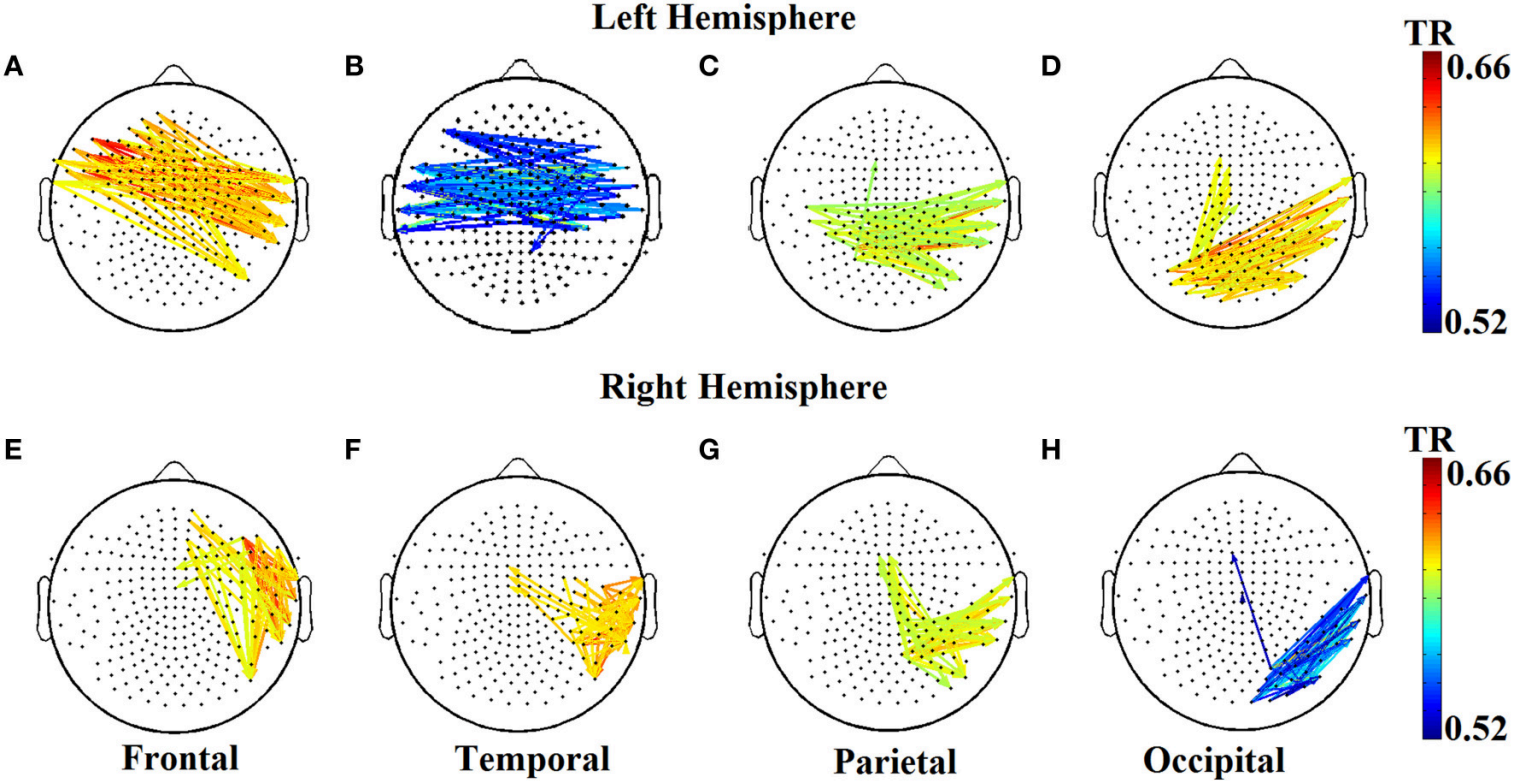
**Group-averaged transition rate (TR) for pairs of sensors located roughly over the four lobes (A–D and E–H) in each hemisphere for RD children**. For better visualization results were thresholded based on mean +2SD from the set of 248 × 248 TR values in order to reveal the most significant TRs over the entire sensor space. Corresponding TR values failed to reach significance in the NI group (not shown).

**Figure 6:**
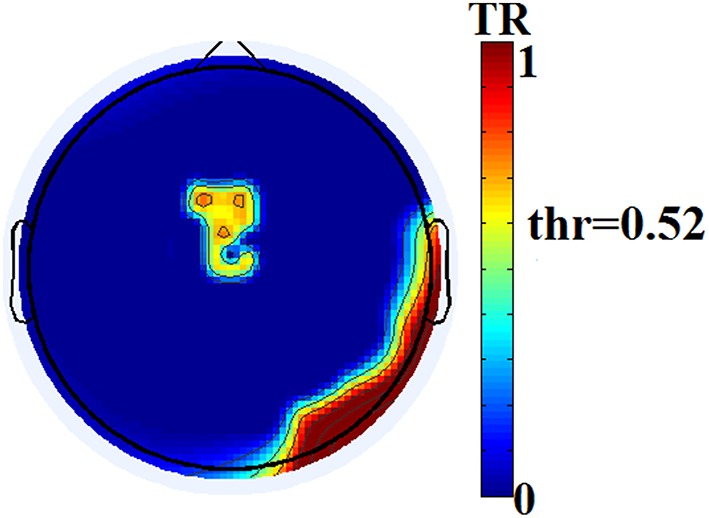
**Group-averaged distribution of transition rates (TR) for within-sensor PAC-interactions in the group of RD participants**. Thresholding (based on mean +2 SDs of the 248 TR values) has been applied revealing sensors located over right hemisphere temporo-parietal and occipito-temporal regions and the midline, that show maximum instability of local PAC interactions.

The two reading groups differed not only on the cross-frequency distributions of prominent PAC modes but also on their stability over time. TR values (between and within sensors) were near zero for the majority of NI children exceeding the threshold of ~0.003 (corresponding to 2 transitions across steps of the sliding windows) in only three participants. In contrast, TRs frequently exceeded the empirically-derived threshold in RD children. The topographic distribution of between-sensor TR values was visualized by applying a threshold of (mean + 2 SDs) to the set of 248 × 248 TRs in the group-averaged TR data. Figure 5 shows sensor pairs associated with “significant” TR values topographically organized by “lobe” and hemisphere. The figure indicates significant temporal variability in the CFC interactions driving right hemisphere brain oscillations by (lower frequency) left hemisphere brain rhythms. Temporally variable CFC interactions driven by right hemisphere oscillations were restricted within the same hemisphere. Significant temporal variability of within-sensor CFC interactions was also more prominent at right hemisphere sensors as shown in Figure 6.

### Dynamic information exchange rate

**Figure 7:**
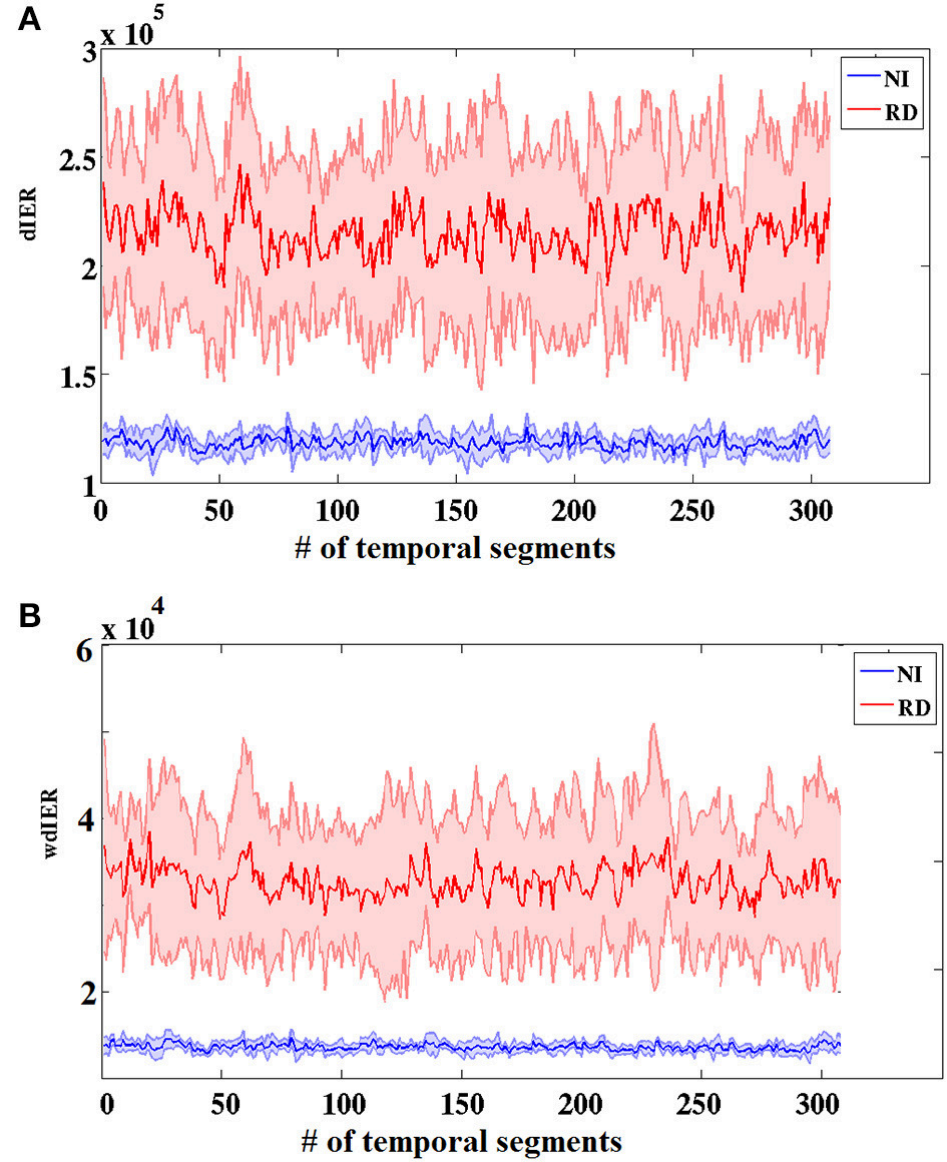
**Group-averaged dynamic (dIER; A) and weighted dynamic (wdIER; B) Information Exchange Rate showing significant differences between the RD and NI groups (Wilcoxon score, *p* < 10^−11^)**. Value sums over the entire network (between every possible pair of sensors) are shown on the vertical axis. Thinner lines indicate ± 1 SD intervals.

As expected on the basis of the aforementioned group differences on TR values, the RD group showed significantly higher values on both global measures of dynamic information exchange rate (dIER and wdIER; Wilcoxon test, *p* < 10^−12^, Figure 7).

### Classification of RD and NI readers based on dynamic information exchange rates

Results of each of the adopted cross-validation schemes indicated perfect discrimination between the two groups based on both dIERs and wdIERs profiles (see Section 8 in Supplementary Material). Similar results (i.e., 100% classification accuracy) were obtained when using the 28D vector from the individual probability distribution (PD) of prominent PAC modes (PD^PAC^) and also the TR profiles (see Figure 4). For comparison, classification accuracy based on relative power in the same frequency bands used to estimate CFC did not exceed 70% (see Section 2 in Supplementary Material).

### Associations between TR values and cognitive measures

**Figure 8:**
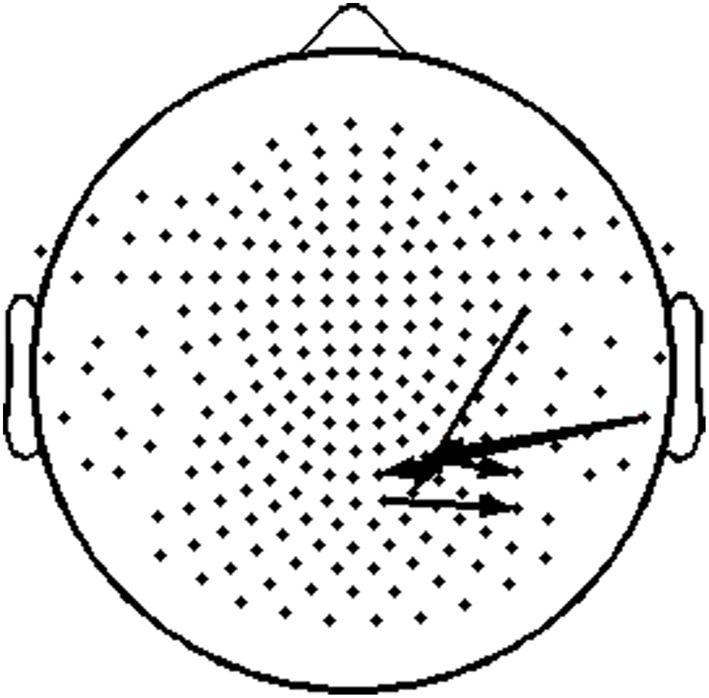
**Topography layout of the five pairs of sensors, located over right temporo-parietal regions, where significant positive correlations were found between TR values and reading measures among RD participants**.

To assess the functional significance of indices of dynamic information exchange developed in the present work, we computed partial correlations between reading achievement scores and TRs (adjusting for age) computed for pairs of sensors where significant group differences were detected (sensors located over right temporo-parietal areas). Significant correlations, restricted to the RD group for WJ3-Reading Composite scores, were found at five sensor pairs, the topography of which is shown in Figure 8. Significant partial correlations between reading achievement/IQ measures and TR values from the five sensors located over right temporo- parietal sensors are tabulated in Table 2.

**Table 2:** **Significant partial correlations between reading achievement/IQ measures and TR or PD values at right temporo- parietal sensors (RD group)**.

		**Composite**	**LWID**	**WA**	**VIQ**	**PIQ**
Sensor pair (TR values)	113–143	0.51^†^	0.47^*^	0.44	0.45^*^	0.46^*^
	108–191	0.56^†^	0.39	0.40	0.49^*^	0.45^*^
	138–208	0.50^†^	0.42	0.43	0.43	0.40
	245−106	0.54^†^	0.45^*^	0.41	0.39	0.37
	146−139	0.52^†^	0.41	0.39	0.43	0.39
Frequency pair (PD values)	δ–θ	0.67^†^	0.49^†^	0.53^†^	0.48^†^	0.58^†^
	θ–γ	0.74^†^	0.53^†^	0.67^†^	0.71^†^	0.49^†^
	α2–γ	0.75^†^	0.63^†^	0.61^†^	0.69^†^	0.63^†^

*Abbreviations, LWID, WA, Composite, Woodcock-Johnson III Letter-Word Identification, Word Attack, and Basic Reading composite, respectively; VIQ, WASI Verbal IQ score; PIQ, WASI Performance IQ score*.

† *p < 0.01*,

* *p < 0.025*.

### Associations between PD values and cognitive measures

**Figure 9:**
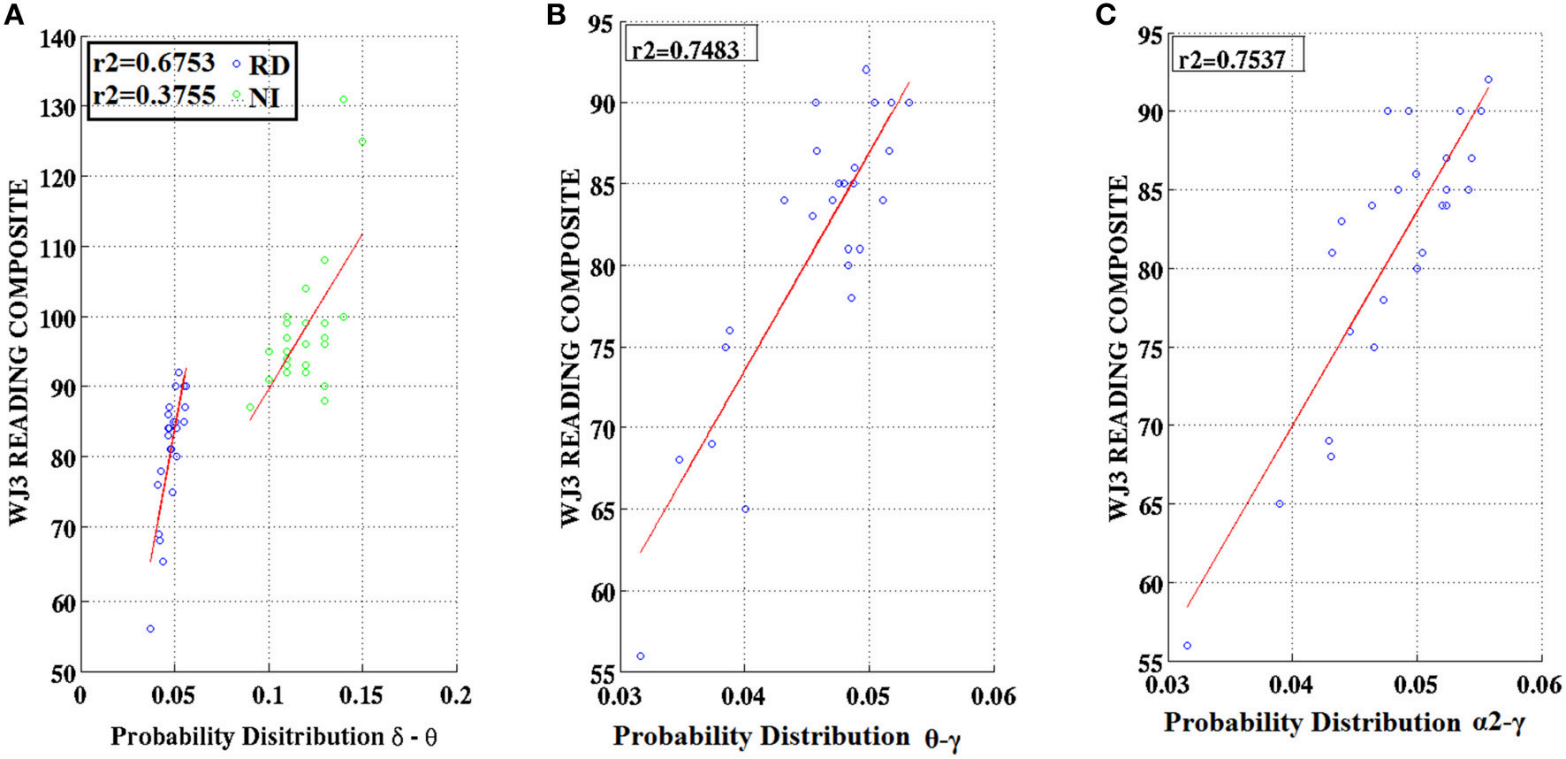
**Regression scatter plots of Woodcock–Johnson III (WJ3) Reading Composite scores over Probability Distribution values for three frequency pairs [(A) δ-θ, (B) θ-γ, (C) α2-γ] aggregated over right temporo-parietal sensors among RD participants (blue circles; B,C)**. A significant association noted for NI participants (green circles) is included in **(A)**.

**Figure 10:**
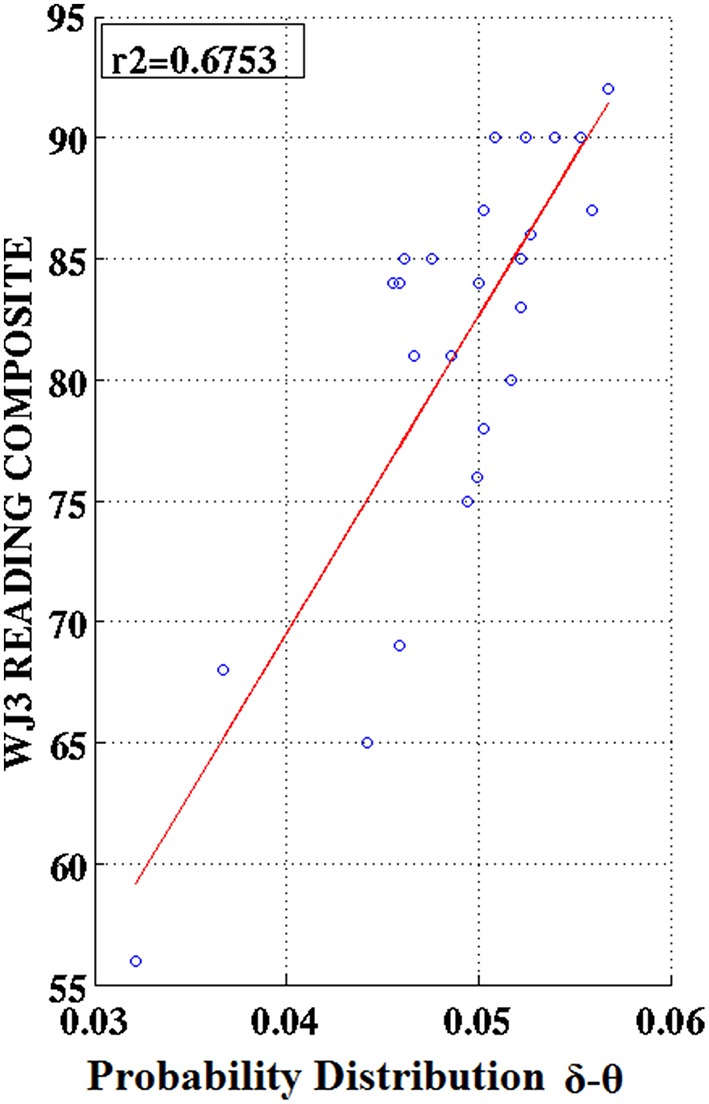
**Regression scatter plots of Woodcock–Johnson III (WJ3) Reading Composite scores over Probability Distribution values aggregated over right temporo-parietal sensors among RD participants**.

Partial correlation coefficients (adjusting for age) were computed between PDs integrated over time and sensors located above right temporo-parietal areas and reading achievement/IQ scores. Significant positive associations (*p* < 0.01) were found between WJ3-Reading Composite scores and PD values for three frequency pairs: δ-θ, θ-γ, and α2-γ which were restricted to the RD group (Figures 9, 10). Among NI participants there was a moderate-size correlation (*r* = 0.61) which, however, did not reach significance (Figure 9A). Significant partial correlations between reading achievement/IQ measures and PD values for the three frequency pairs are tabulated in Table 2.

### Predictability of dynamic information flow based on dominant cross-frequency pairs

**Figure 11:**
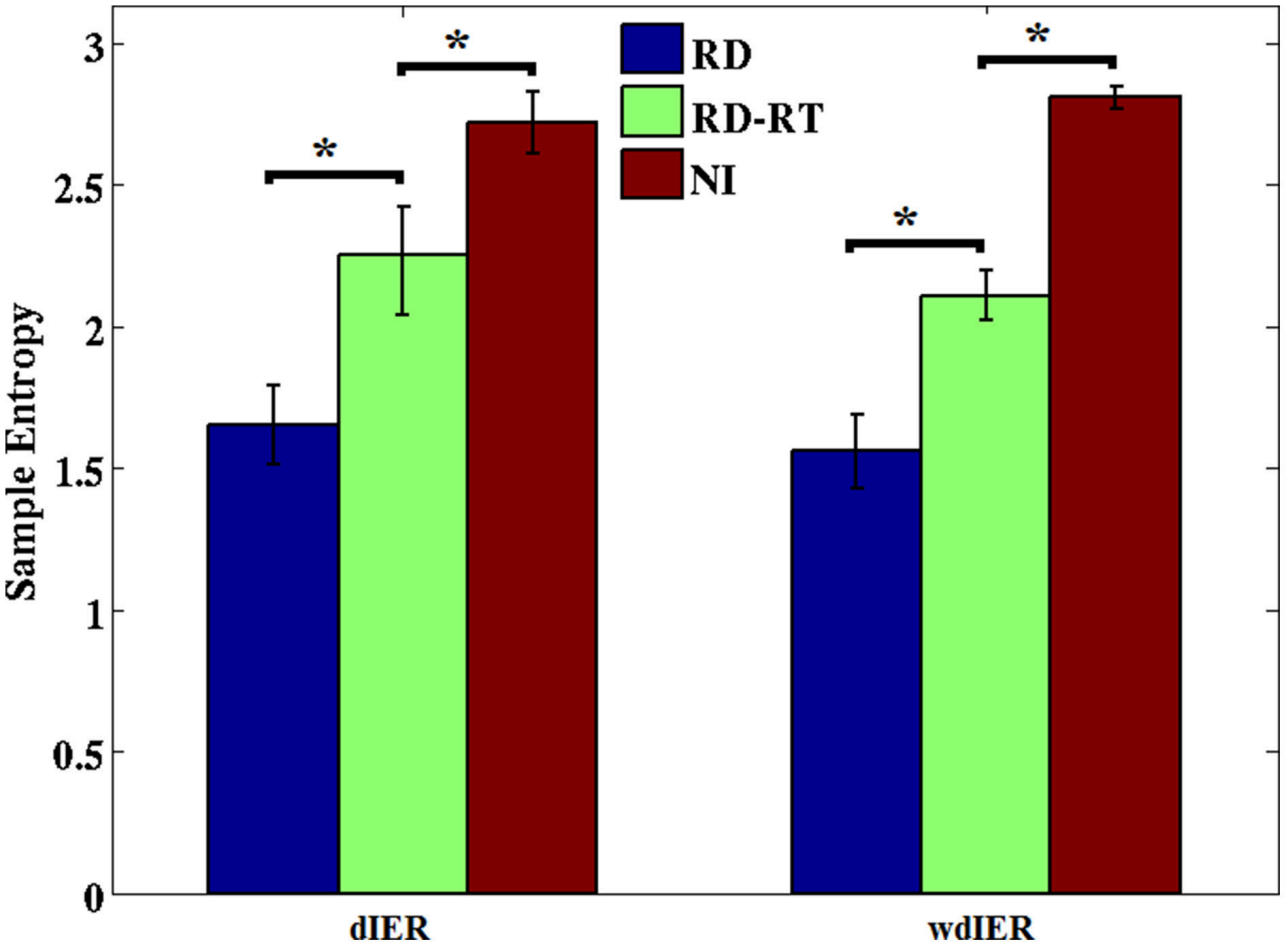
**Average entropy estimates derived from dIER and wdIER values aggregated across all sensors for the RD (blue bars) and NI groups (red bars)**. Notice the significant increase in entropy in the RD group after excluding sensors located over right temporo-parietal regions (RD-RT; green bars). ^*^p < 0.001, Wilcoxon rank-sum test. Standard error in bars.

Sample entropy values estimated over dIER and wdIER were significantly higher for NI as compared to the RD group implying lower overall predictability of CFC interactions in the latter group (*p* < 0.001, Wilcoxon rank-sum test; see Figure 11). Interestingly, there was a significant increase in entropy values in this group, when estimated for sensors located over the right temporal brain region (where the highest transition rates were originally observed).

## Discussion

Here we report results from a novel analytic approach to study resting-state functional connectivity derived from neuromagnetic data in sensor space. The first key finding is that the prominent mode of phase-amplitude coupling between neuronal assemblies in RD children at rest involves rhythmic oscillations that vary widely in frequency. In contrast, the typical mode of CFC interactions among NI readers involved driving the amplitude of a particular brain oscillation by the phase of oscillatory activity that differed very little in frequency from the former. The second notable finding is that the spectral mode of these interactions was highly stable among typically-achieving readers during the 3-min recording session. In contrast, cross-frequency interactions in RD students showed high levels of temporal instability and lower levels of entropy (predictability). Correlational analyses revealed positive associations between indices of dynamic CFC interactions in recordings over right hemisphere temporo-parietal brain areas and reading achievement scores among struggling readers. The proposed dynamic connectomic features (dIER, wdIER, TR, and PD^PAC^) appeared sufficient to discriminate NI from RD students with a high degree of accuracy.

### Repertoire of CFC modes

Phase-amplitude phenomena, also referred to as “nested oscillations,” occur when the amplitude of an oscillation at a particular frequency is modulated by the phase of a lower-frequency oscillation. This phenomenon has been demonstrated in various species and brain areas and is postulated to be an important attribute of the coordinated activity taking place in distinct neuronal populations, ensuring efficient transfer of information (i.e., signaling that is accurate in timing and high in gain; Varela et al., 2001; Buzsáki, 2006; Buzsáki and Watson, 2012; Buzsáki et al., 2013). Such functional connectivity patterns that characterize resting activity may serve as the dynamic substrate of complex cognitive functions, such as reading, which involving multisensory integration and coordination of several distinct component operations (visual/graphemic processing and recognition, phonological processing, and articulation).

Different oscillation frequencies are characterized by different spatial scales for the interacting neuronal populations, with lower frequencies having a higher capacity to modulate activity in remote brain regions than higher frequencies (von Stein and Sarnthein, 2000). The spectral separation between the two oscillating frequencies may also be important for efficient information exchange (Buzsáki and Watson, 2012) given that, at last in principle, the number of cycles of the higher frequency encapsulated within the phase of the slower frequency is related to the amount of information being exchanged between the two oscillators (Jensen and Colgin, 2007; Canolty and Knight, 2010; Florin and Baillet, 2015). Cross-frequency phase-to-amplitude coupling may have evolved as a compensatory mechanism that enables efficient information transmission without the need to slow-down the processing rate of the receiving neuronal population to match that of the signal source (Buzsáki and Watson, 2012; Buzsáki et al., 2013). Presently we can only surmise that the substrate of relatively stable CFC-related dynamic information exchange observed among children with normal developmental histories reflects a typical and, presumably, optimal working level ensuring efficient neuronal transmission (Deco and Corbetta, 2011; Deco et al., 2013).

### Temporal variability of CFC profiles

An example of the increased temporal variability of CFC profiles characterizing RD participants, is that for a given pair of sensors A, B the amplitude of the neuromagnetic signal in the β1 frequency band in sensor B may be initially driven by the phase of δ oscillations in sensor A. During subsequent time intervals, activity in sensor B may be characterized by the driving of γ amplitude by α_1_ phase in sensor A whereas, only seconds later, α_2_ amplitude in sensor B may be driven by the phase of θ oscillations in sensor A. The same was true for cross-frequency interactions at each sensor separately. This type of temporal variability was summarized by global indices of temporal variability in CFC interactions (dIER and dwIER profiles) which clearly differentiated NI from RD students with very high levels of accuracy in the context of different, complementary cross-validated classification schemes. A complementary, global index of predictability in CFC interactions, namely entropy, corroborated these findings by demonstrating higher randomness in the recordings obtained from RD participants.

Importantly, the patterns of temporal variability in cross-frequency interactions among struggling readers showed distinct topographies. PAC modes derived from activity recorded by each sensor separately were indicative of highly unstable cross-frequency interactions between neuronal assemblies located in right hemisphere temporo-parietal and occipito-temporal regions. Between-sensor analyses complemented this finding, suggesting the presence of highly variable CFC interactions of neuronal assemblies distributed within the right hemisphere (e.g., between frontal-temporal, and between temporal-occipital regions). Significant temporal variability was also found in the CFC interactions between left hemisphere occipital, frontal, and temporal sites “driving” higher frequency oscillations in corresponding right hemisphere sensors. Entropy findings further point to right temporo-parietal sensors in RD students as showing the highest levels of randomness in CFC interactions in the entire sensor space.

Although firm conclusions regarding the functional significance of these findings cannot be drawn at present, results raise a number of intriguing possibilities regarding the relevance of ongoing patterns of neuronal coupling at rest for task-related engagement of cortical areas during reading in RD. Overexitability of right hemisphere areas at rest may account for previous reports concerning task-related overactivation of right hemisphere “reading” areas in this population (Pugh et al., 2001; Milne et al., 2002; Hoeft et al., 2007, 2011; Simos et al., 2011). Both the extended repertoire of PAC interactions and their pronounced temporal variability may suggest an inefficient substrate for neuronal communication within and between right hemisphere regions. Correlational analyses support this notion by revealing positive associations between both the degree of temporal variability and the strength of temporally aggregated CFC interactions over right temporo-parietal sensors and reading achievement scores in the group of struggling readers. The fact that positive associations were found implies a compensatory mechanism, which is nevertheless not sufficient to support efficient reading ability. Moreover, the relative specificity to reading measures suggests a more prominent role of this mechanism for reading (as opposed to more general language or cognitive ability). The frequency range of CFC interactions associated with these significant correlations (δ-θ, θ-γ, and α2-γ) suggests a pattern of spectrally widespread alterations in the resting-state substrate of functional interactions in the RD group. These results complement our previous finding suggesting increased local efficiency of cortico-cortical interactions over left temporo-parietal regions in typical as compared to struggling readers revealed by resting-state synchronization measures (Dimitriadis et al., 2013b). Interestingly, the positive correlations between left hemisphere synchronization efficiency indices and reading achievement were restricted to the group of typical readers.

In more general terms, these results are consistent with an emerging view in cognitive neuroscience that the brain is a self-organizing complex system which, under normal circumstances, displays a limited range of variability of spatiotemporal configurations (Sporns et al., 2004; Shanahan, 2010; Deco and Corbetta, 2011; Tagliazucchi et al., 2012; Carhart-Harris et al., 2014; Hansen et al., 2015; Dimitriadis et al., 2015a). Higher levels of variability and temporal instability of signaling modes, such as those characterizing the neuromagnetic activity of RD students at rest, may signify a critical level of function. Under certain conditions, for instance when struggling readers are required to perform more demanding, yet age-appropriate reading tasks, the extensive brain network that is normally responsible for decoding and word recognition is more likely to operate in a transition zone between order and chaos, resulting in suboptimal performance (Tononi et al., 1994; Shanahan, 2010; Deco and Jirsa, 2012; Tagliazucchi et al., 2012).

### Limitations of the study

The present results should be considered in light of a number of methodological limitations. First and foremost functional connectivity estimates were obtained at the sensor level therefore limiting the type of conclusions that can be drawn regarding the efficiency of interactions between specific, underlying neuronal populations. Moreover, volume conduction effects can produce artificial results even in the case of CFC, which is considered to be less susceptible to volume conduction effects (Engel et al., 2013). However, several steps were taken in order to minimize the effect of volume conduction in the calculation of CFC estimates, namely transformation of axial gradiometer data to planar, orthogonalization of the signals and use of the imaginary portion of PLV. The success of these measures is indicated by negligible correlations between PAC time-series computed between adjacent sensors (see Figure S3 in Supplementary Material). Failure to find substantial correlations between relative power and PAC strength in the temporal domain (see Figure S4 in Supplementary Material) lends further support to the claim that our results were not contaminated by volume conduction.

A second issue to be considered when interpreting the current results concerns the sensitivity to low-frequency phase components with asymmetric cycles, a phenomenon noted in intracortical recordings (Steriade et al., 1996; Buzsáki and Draguhn, 2004; Haider et al., 2006). Moreover, edges in the data may cause spurious PAC phenomena (Kramer et al., 2008). It should be noted however that PAC has been found in numerous studies using ECoG, LFP, and MEG data (Canolty et al., 2006; Tort et al., 2010; Özkurt and Schnitzler, 2011) making it unlikely that this phenomenon is artifactual. The application of very steep bandpass filters (Third order forward and backward Infinite Impulse Response filter) served as an additional step in the present study against the likelihood of spectral cross-contamination between adjacent frequency bands. Moreover, the spectral separation between the low (modulating) frequency and the frequency associated with the highest power in the signal was probably sufficient to rule out the possibility that PAC phenomena where induced by high-power low frequency oscillations dominating the resting state recordings.

### Strengths of the study and future directions

Despite these limitations, the present study has a number of novel features. The main strength of the study lies in the implementation of a dynamic functional connectivity methodology (Dimitriadis et al., 2009, 2010a,b, 2012a,b, 2013a,b, 2015a,b,c; Deco et al., 2011, 2013; Damaraju et al., 2014; Kopell et al., 2014) to explore how resting-state is shaped by predominant cross-frequency coupling (Jirsa and Müller, 2013; Florin and Baillet, 2015). This methodology was adapted to MEG data that are uniquely suited to provide estimates of instantaneous information exchange between neuronal oscillators affording the requisite subsecond temporal resolution.

Although analyses were conducted in sensor space, MEG recordings were first converted to planar gradiometer field approximations, ensuring that the maximum signal lies directly above active brain areas. Moreover, our method represents a step forward from traditional investigations of functional connectivity, by taking into account temporal variability in cross-frequency coupling (Dimitriadis et al., 2009, 2010a,b, 2012a,b,c, 2013a,b, 2015b, 2016; Deco et al., 2011, 2013; Damaraju et al., 2014). Importantly for future applications, PAC indices appeared to be both sensitive and selective to the presence of developmental reading difficulties, and were associated with very effective discrimination of RD from age-matched typical readers. In contrast, relative power measures were substantially less effective in discriminating participants from the two groups.

It would be interesting in the future to study both NI and RD participants with the same methodology during experimental paradigms tailored to dyslexia and to compare the topologies, the extracted PAC-oriented markers and the information transmission between rest and cognitive tasks. Moreover, a source localization strategy would improve interpretability of the findings (Ioannides et al., 2012; Florin and Baillet, 2015) in relation to indices of anatomical connectivity DTI (e.g., Richards et al., 2015). At a more basic level, it is important to establish developmental trends in the CFC (and especially PAC) resting-state patterns and examine their functional significance through brain-behavior associations.

To conclude, resting-state phase-amplitude coupling metrics developed in the present study are some of several potentially useful indices of atypical functional brain organization in developmental reading disability. Their functional significance, specificity to component reading processes, which are known to be underdeveloped in struggling readers, and their sensitivity to educational interventions requires further systematic investigation, before their value as diagnostic or prognostic tools can be fully evaluated.

## Author contributions

Conception of the research: SD, PS, JF, AP; Methods design and data analysis: SD; Drafting the manuscript: SD, NL; Critical revision of the manuscript: PS, JF, AP; Every author read and approved the final version of the manuscript.

### Conflict of interest statement

The authors declare that the research was conducted in the absence of any commercial or financial relationships that could be construed as a potential conflict of interest.
