# Supplementary material for: Greater Repertoire and Temporal Variability of Cross-Frequency Coupling (CFC) Modes in Resting-State Neuromagnetic Recordings among Children with Reading Difficulties
Source: Front Hum Neurosci. 2016 Apr 26;10:163. doi: 10.3389/fnhum.2016.00163 (PMC4844915; doi:10.3389/fnhum.2016.00163)
Supplement: Supplementary file 1 [file DataSheet1.PDF]

Supplementary Material

Greater repertoire and temporal variability of cross-frequency coupling (CFC) modes in resting-state neuromagnetic recordings among children with reading difficulties

S.I. Dimitriadis[1,2], N.A. Laskaris[1,2], P.G. Simos[3], J.M. Fletcher[4] and A.C. Papanicolaou[5,6]

[1] Artificial Intelligence and Information Analysis Laboratory, Department of Informatics, Aristotle University, Thessaloniki, 54124, Greece

[2] Neuroinformatics Group, Department of Informatics, Aristotle University, Thessaloniki, Greece / http://www.neuroinformatics.gr

[3]School of Medicine, University of Crete, Greece

[4]Department of Psychology, University of Houston, Houston, Texas, 77204 USA

[5]Department of Pediatrics, Division of Clinical Neurosciences, University of Tennessee Health Science Center, Memphis, TN, USA

[6]Neuroscience Institute, Le Bonheur Children's Hospital, Memphis, TN, USA

Running title: Neuromagnetic Cross-Frequency Coupling in Dyslexia

Address Correspondence to:

Artificial Intelligence and Information Analysis Laboratory, Department of Informatics, Aristotle University, Thessaloniki, 54124, Greece

email: stidimitriadis@gmail.com, Tel: +30-6944-834186 , 30-2310-998706. Fax: +30-2310-998453

Section 1. Estimation of frequency with maximal power

For each participant and MEG sensor we calculated the power spectral density using the Fast Fourier Transform employing partially (50%) overlapping Hanning windows each consisting of 4096 data points. This yielded a frequency resolution of 0.25 Hz. For each window we estimated the modulating frequency associated with the prominent CFC interaction (max PAC value) and independently, the frequency associated with maximum power. If the two frequencies were identical this would imply that the observed prominent CFC interaction was driven by the power of the dominant frequency and not its phase. In order to ensure that we did not include CFC interactions of this type in further analyses, we required a minimum frequency difference of 1Hz between the two frequencies (frequency of the low-frequency phase, frequency of the higher power).

Section 2. Relative Power

Pelative power (RP) was calculated to assess the relative contribution of several oscillatory components to the global power (Leuchter et al., 1993; Rodriguez et al., 1999). RP was computed at each sensor in the following conventional frequency bands: δ (0.5–4 Hz), θ (4–8 Hz), α1 (8–10 Hz), α2 (10–13 Hz), β1 (13–15 Hz), β2 band (15–19 Hz), β3 (20–29 Hz), and γ (30–45 Hz). Group differences were assessed with the Wilcoxon Rank-sum test at each sensor and frequency band (tested at Bonferroni corrected $p < 0.0001$). Topographies of group-averaged RP are shown in Fig. S.1 with white circles indicating sensors where significantly different RPs were found. Results revealed significantly higher δ and θ power at left frontal and higher δ power at right parieto-occipital sensors among NI as compared to RD students. The latter group demonstrated higher θ and α1 power at right temporal sensors, θ power at patieto-occipital sites bilaterally, and α1, α2 and β1 power at left frontal sites. They also showed higher β1 and β2 power at mid-frontal sensors, β2 power at left frontal and parieto-occipital sites, and γ power at right parieto-occipital sites.

Individual classification capacity was tested on Laplacian scores (LSFr_S) as features computed for each of the 8 (frequency bands; Fr) x 248 (sensors; S) RP values, over 5 cross-validation runs. The null distribution for each of the 1984 features was obtained through bootstrapping by randomizing the labels assigned to each feature for 100.000 times. Next, we tested if each the $LS_{Fr\_S\ values}$ of each feature deviated from the null distribution $LS^{R}_{Fr\_S}$ and a (one-sided) p-value was assigned as the percentage of $LS^{R}_{Fr\_S}$ that exceeded the original estimated LS_{Fr_S}. Next, the obtained p-values were Bonferroni-

corrected ($p < 0.05/(8*248)$). Finally, the data were submitted to a k-NN (k-nearest neighbor) classifier using the majority vote criterion. For comparison purposes, we also employed a linear Support Vector Machine (SVM) classifier. Table S1 summarizes classification performance, specificity, sensitivity, and the number of features employed by frequency band.

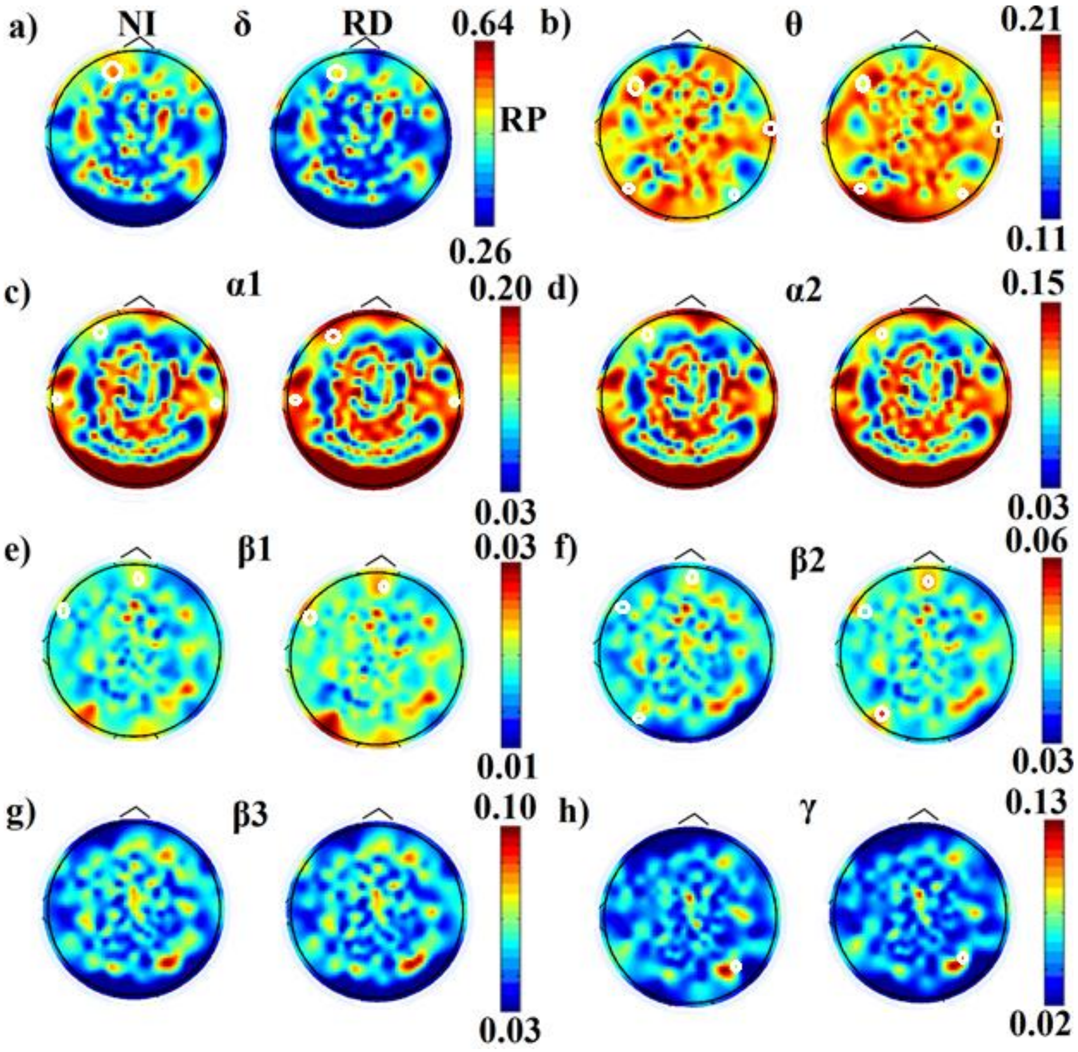

Figure S1. Average topographies of relative power in each frequency band for non-reading impaired (NI) and students with reading difficulties (RD). Sensors where significant RP differences were found between groups are shown as white circles (Wilcoxon Rank-sum test evaluated at Bonferroni corrected $p < 0.0001$).

Table S1. Classification performance of the k-NN and linear (SVM) classifiers (average of 5 runs).

k-NN classifier			Linear SVM			
**CP%**	**Sensitivity %**	**Specificity %**	**CP%**	**Sensitivity %**	**Specificity %**	**Number of Features δ-θ-α1-α2-β1-β2-γ**
67.25±8.72	65.35±6.13	65.94±9.35	70.16±10.54	65.76±9.15	67.04±9.54	4-3-1-2-3-0-1

Section 3. Alpha Peak Frequency

Alpha peak frequency was computed for each participant by averaging across sensors and across the 6 30-sec MEG segments (Klimesch, 1999). In participants lacking a distinct α peak we used the gravity frequency (f(i)), which was calculated as the weighted sum of spectral estimates, divided by α power:

$$f(i) = (\sum (a(f) * f)) / (\sum (a(f)) ,$$

where a(f) denotes power spectrum estimates at frequency f. The index of summation is in the 7–13 Hz range. Results showed that the two groups had comparable α peak frequencies (NI group: Mean = 10.76, SD = 0.031; RD group: Mean = 10.71, SD = 0.34; Wilcoxon Tank-Sum Test, $p < .001$).

Section 4. Probability distribution of prominent frequency pairs across space and time

Fig. S2 complements Fig. 4 by displaying average probability distributions (PD) aggregated separately over time segments (a, c) and sensor pairs (b, d). The graphs are very similar to those displayed in Fig. 4 where data were aggregated over both time segments *and* sensor pairs.

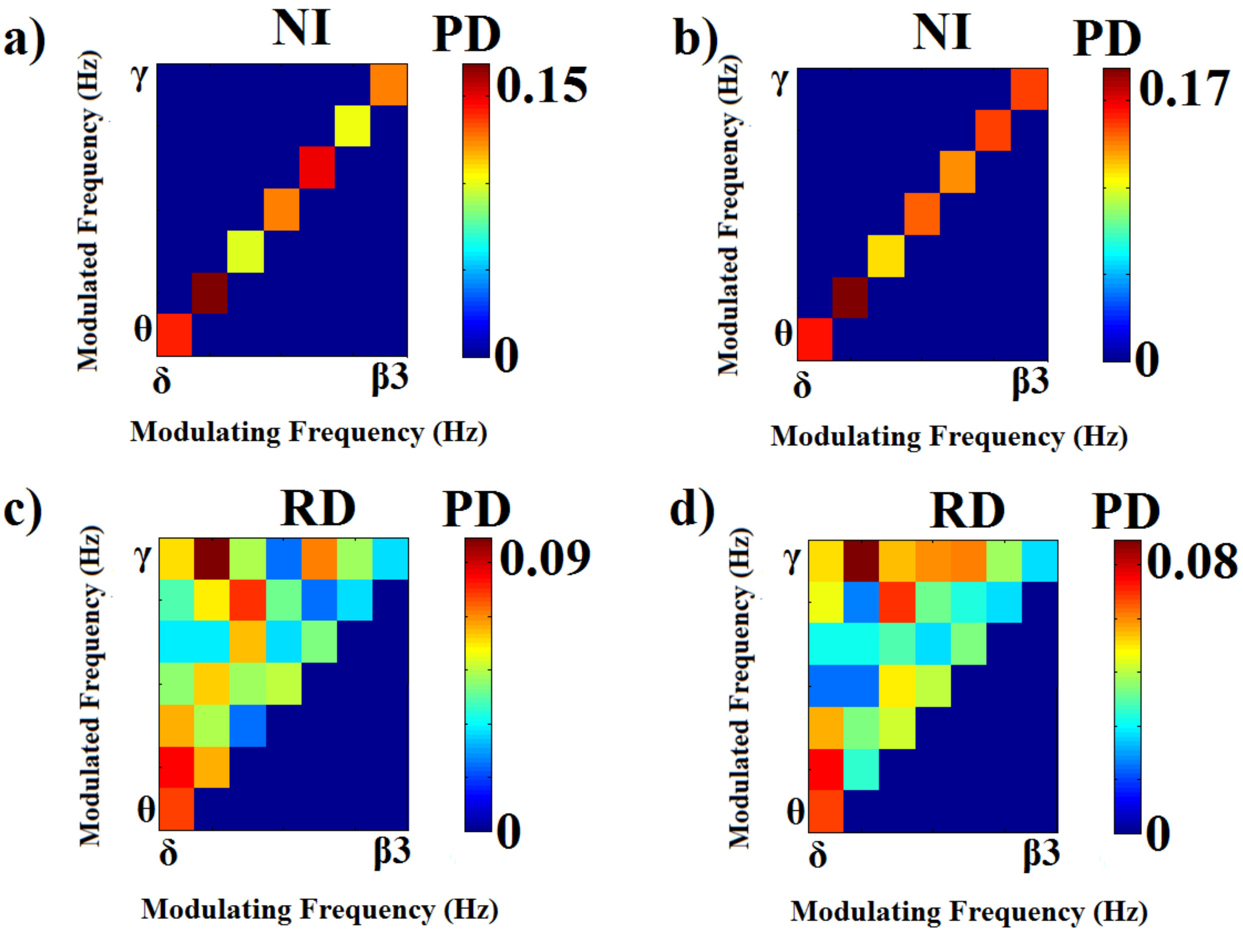

Figure S2. Group-averaged empirical Probability Distribution profiles, aggregated over time (a, c) and over sensor space (b, d), for non-impaired (NI) and struggling readers (RD).

Section 5. Evolution of PAC strength as a function of prominent CFC pair

Fig. S3 demonstrates the evolution of PAC strength as a function of the corresponding modulating frequency for three neighboring sensor pairs located above the left frontal lobe in a RD participant. Both PAC strength and the integrated probability distribution (PD) for the three pairs clearly demonstrate a separate uncorrelated functionality which further supports the validity of our methodology. The cross-correlations of the PAC time series between the three pairs of sensors averaged 0.16 ± 0.05 which further supports the spatial independence of PAC indices.

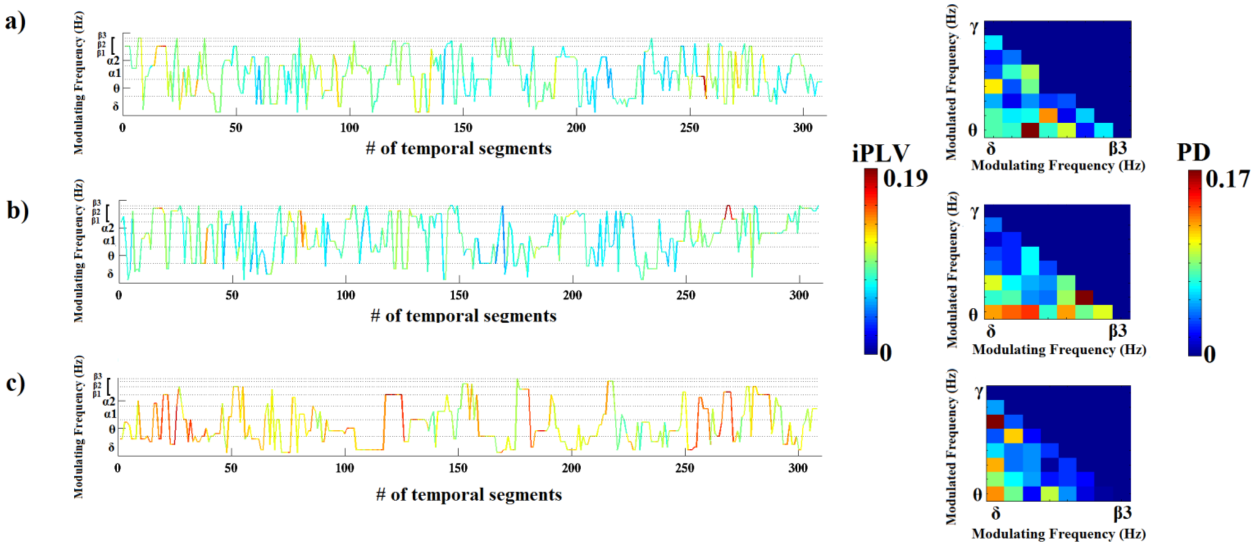

Figure S3. The evolution of PAC strength as a function of the frequency of the modulating oscillation (y axis) over time for three pairs of sensors (a-c) located over left frontal brain areas from a RD participant. Color denotes the strength of iPLV. The rightmost matrices summarize the probability distribution (PD) of prominent PAC pairs aggregated over time for each pair of sensors.

Section 6. Evolution of PAC strength with Relative Power

Examples of the evolution of PAC strength along with corresponding relative power (RP) of the modulated frequency are shown in Fig. S4 for 3 neighboring pairs of sensors located over the left frontal lobe in two randomly selected participants (RD: upper panel and NI: lower panel). The weak correlation of PAC strength with RP over time (r = -0.12 ± 0.06 across participants and sensors) supports the independence of the two measures.

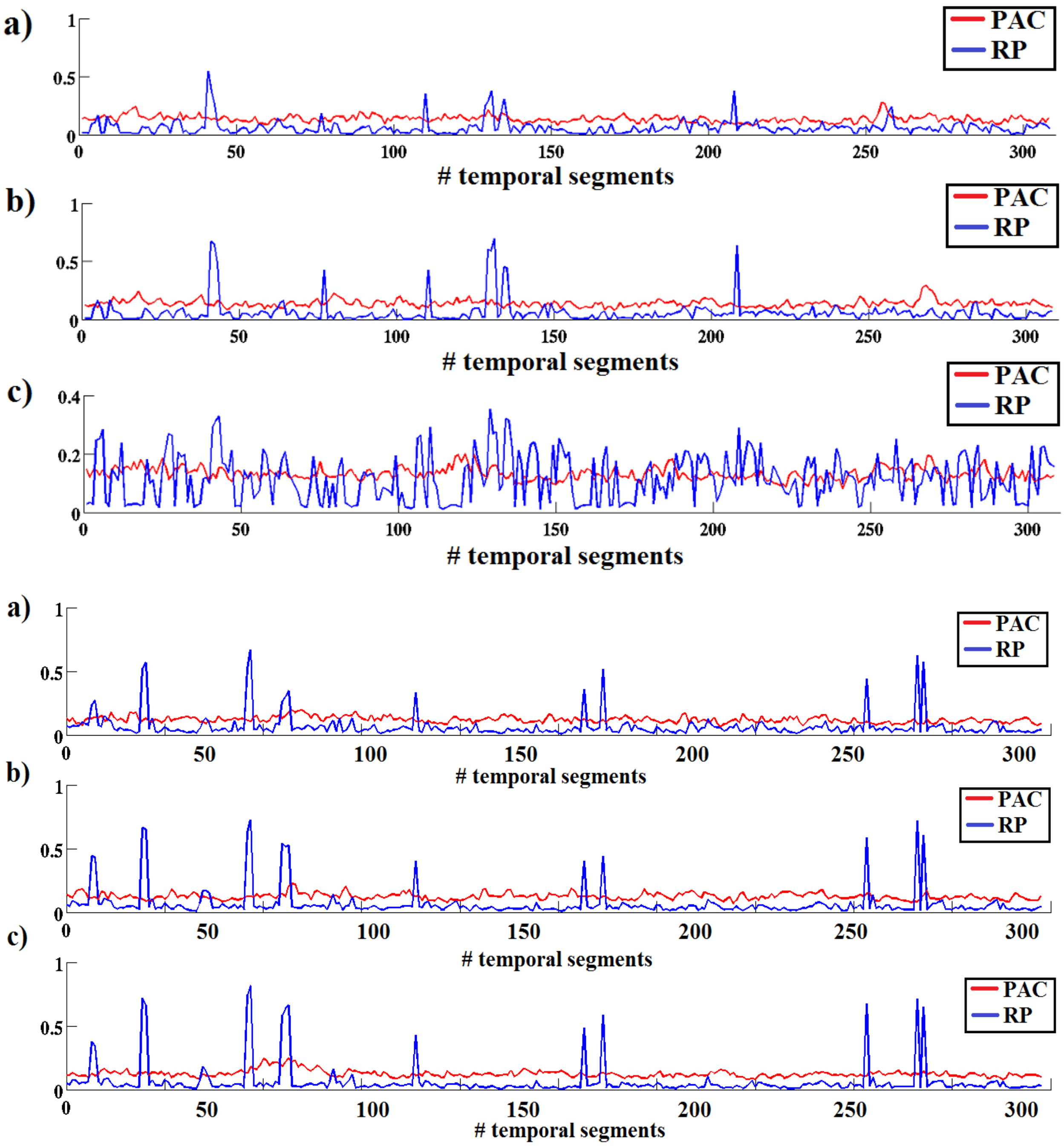

Figure S4. Evolution of PAC strength and relative power (RP) of the modulated frequency for three neighboring pairs of sensors (a-c) located over the left frontal lobe in a randomly selected RD (upper panel) and a NI participant (lower panel).

Section 7. IER and WIER based on PLV

While PLV is less susceptible to amplitude effects than coherence-based synchrony measures, it can be prone to artificial phase-locking due to volume conductance and common reference (Nolte et al., 2004). For this reason, all analyses reported in the main text were conducted on the imaginary porion of PLV (iPLV), which is only sensitive to non-zero phase lags and thus resistant to instantaneous 'self-interaction' from volume conductance. Group-averaged temporal evolution of both dIER and wdIER as presented in Fig. 7 based on iPLV and Fig. S2 based on PLV further support this approach.

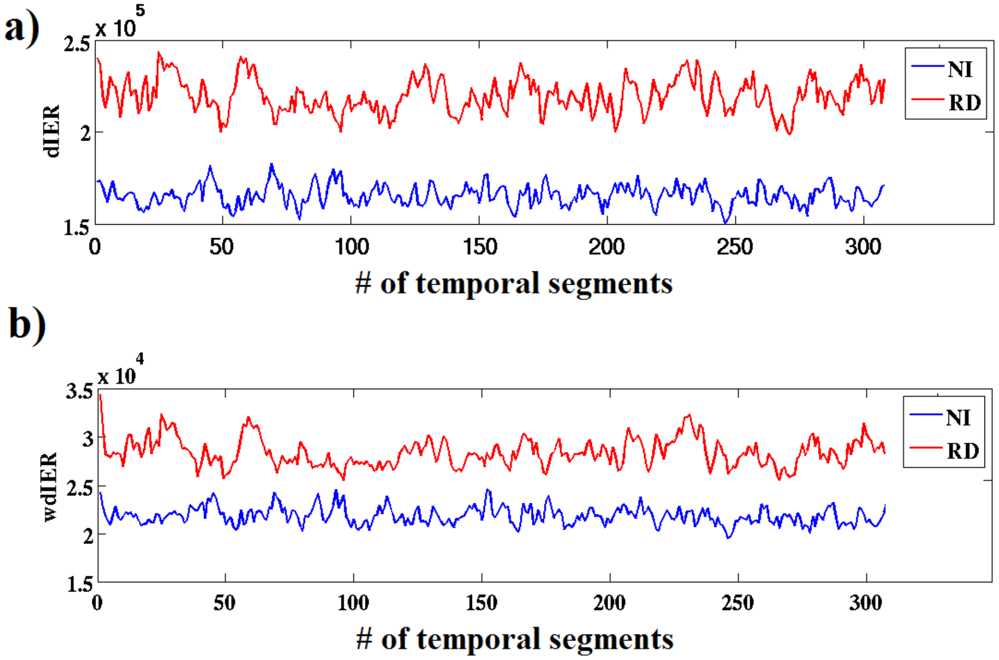

Figure S5. Group-averaged temporal evolution of dIER and wdIER based on PLV.

Section 8. A dissimilarity measure for dynamical trajectories based on the Wald-Wolfowitz (WW) test

The two-sample, non-parametric WW test was adopted in the present work to assess the degree of similarity between two time series of measures (dIER and wdIER) derived from continuous MEG recordings. The procedure entailed, first, transforming each dIER and wdIER time series $x(t)$, $t = 1.2,\ldots T$ into dynamic trajectories represented by multidimensional vectors $X_t = [x(t), x(t+1),\ldots, x(t+d_e)]$ and $Y_t = [y(t), y(t+1),\ldots, y(t+d_e)]$ (X and Y correspond to two participants or to two split-half segments from a single participant). These vectors were formed by selecting the appropriate set of d_e, which is the

embedding dimension parameter that controls the dimensionality of the vectors and d_t is the time-delay. By adopting the Ragwitz criterion, we optimized the embedding dimension *de* and the embedding delay *dt* (Ragwitz and Kantz, 2002), resulting in values ranging from 3 to 6 in both the complete and split-half temporal segments of dIER and wdIER series. The two point-samples $\{X_t\}_{t=1:m}$ and $\{Y_t\}_{t=1:n}$ were then formed and the $w_{dist}= w(\{X_t\},\{Y_t\})$ was computed.

Next the the minimal spanning tree (MST) graph of the overall sample was constructed (i.e., disregarding the sample identity of each point). In this graph points represent nodes with N-1 edges (N = n + m) (i.e., paths within each pair of nodes). The second step of the procedure entails computing the R statistic which is the total number of consecutive sequences with identical sample identities (i.e., “runs”). Based on the number of edge pairs of MST sharing a common node and the degrees of the nodes, the mean and variance of R can be calculated [2]. This property of R permits computation of the initial form of the, normally-distributed, WW Dissimilarity Index (w) as follows:

$$w = \frac{R - E[R]}{\sqrt{Var[R]}}$$

The measure used in classification schemes in the present work was derived from w using the Heaviside step function H(x) as follows: wdist = |w|.H(-w). The higher the value of *wdist*, the more dissimilar the two point-sets are considered to be. Figure S6 visualizes the WW procedure for wdIER data obtained from a NI and an RD participant.

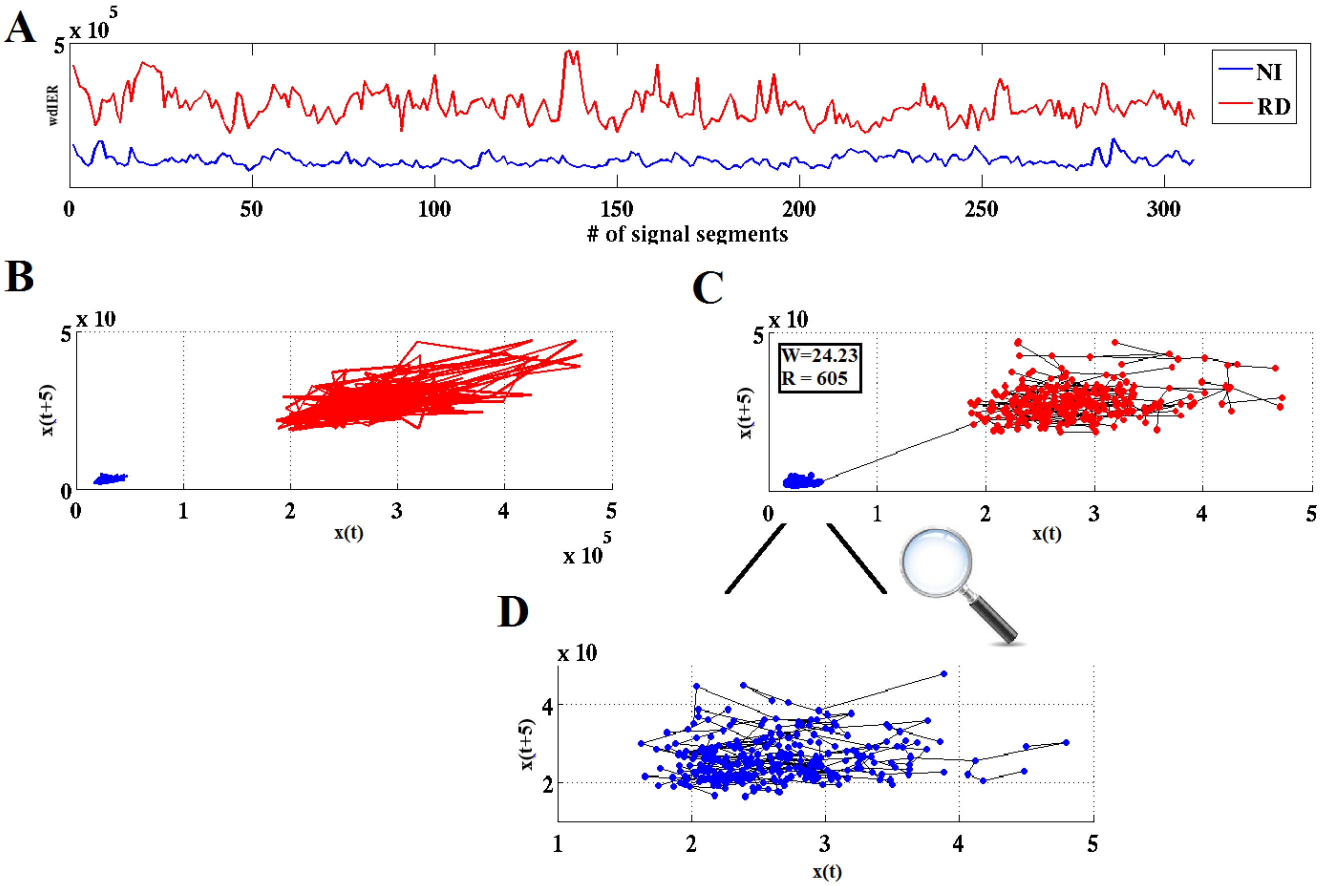

Figure S6. From wdIER-signals to similarity-relations: (a) wdIER for a NI (blue lines) and a RD participant (red lines); (b) superposition of two reconstructed trajectories (time delay = 5, embedding dimension = 3); and (c) using the WW test to assess similarity between the two given trajectories based on the common MST-based edges that connect the two point clouds. (d) Enlarged representation of the MST-based edges for the reconstructed space of wdIER derived from the NI participant shown in (c).

Section 9. Sample Entropy

Like approximate entropy (ApEn), **Sample entropy** (**SampEn**) is a measure of complexity (Richman et al., 2000). But it does not include self-similar patterns as ApEn does. For a given embedding dimension m, tolerance r and number of data points N, SampEn is the negative logarithm of the probability that if two sets of simultaneous data points of length m have distance $< r$ then two sets of

simultaneous data points of length m+1 also have distance < *r*. We defined sample entropy by *SampEn(m,r,N)* or *SampEn(m,r,d_t,N)* where d_t is the time-delay.

Assuming a time-series of length $N = \{x_1, x_2, x_3, ..., x_N\}$ with a constant time interval d_t. We define a template vector of length m, such that $X_m(i) = \{x_i, x_{i+1}, x_{i+2}, ..., x_{i+m-1}\}$ and the distance function $d[X_m(i), X_m(j)]$ (i≠j) like Chebyshev distance (it could be Euclidean distance or any other appropriate distance). At first step, the number of vector pairs in template vectors of length *m* and *m + 1* having $d[X_m(i), X_m(j)] < r$ and denote it by B and A respectively. Then, the sample entropy is defined as :

$$SampEn = -\log \frac{A}{B}$$

Where,

A= no of template vector pairs having $d[X_{m+1}(i), X_{m+1}(j)] < r$ of length *m+1*

B= no of template vector pairs having $d[X_m(i), X_m(j)] < r$ of length *m*

It is clear from the definition that A will always have a value smaller or equal to B. Therefore, *SampEn(m,r,d_t,N)* will be always either be zero or positive value. A smaller value of *SampEn* reflects more self-similarity in data set or less noise.

Generally we take the value of *r* to be 0.2*std where std refers to standard deviation of the time series.

Section 10. Distribution of reading and IQ scores within and between groups.

Fig. S7 presents boxplots of individual reading and IQ scores for RD and NI participants.

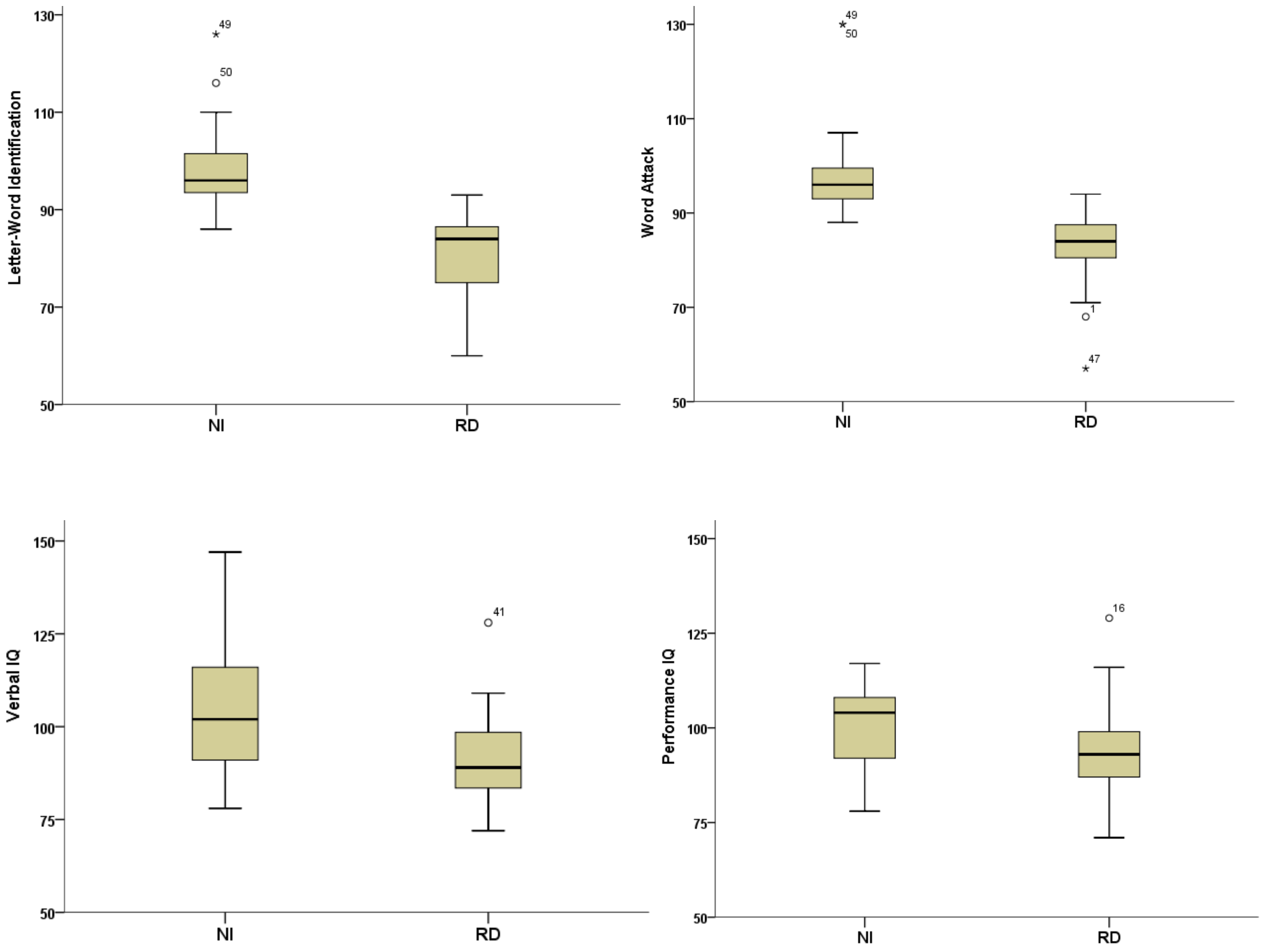

Fig. S7. The distribution of individual participant scores on reading (Woodcock-Johnson III Letter-Word Identification and Word Attack) and IQ measures (WASI Verbal and Performance IQ scores).

Section 11. Definition of a cycle

The phase-plane of a sinusoid harmonic oscillator is a a circle. The period (circle) of this oscillations is related with one running of the unit circle or with this set of up and down of activity over time (**Figure S8**).

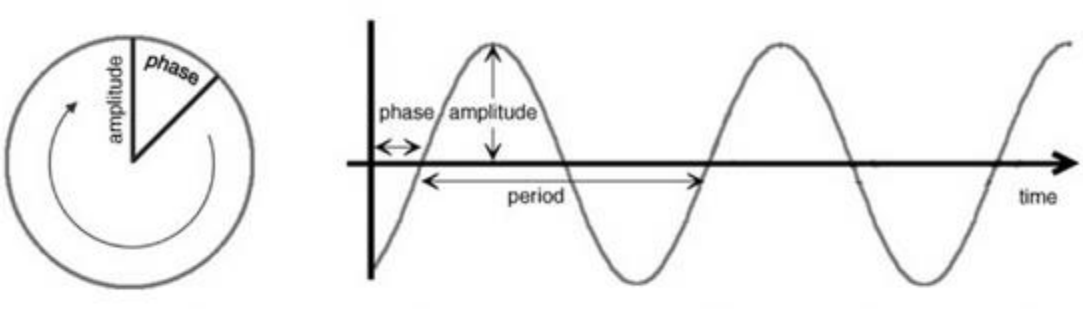

Figure S8. Illustrations demonstrate the orthogonal relationship between frequency and time. An event encapsulated within a brain rhythm can be repeated over time. One period (cycle) corresponds to the perimeter of a circle.

References

[1] Dimitriadis SI**,** Laskaris NA, Tsirka V, Vourkas M, and Micheloyannis S**.** (2010). What does delta band tell us about cognitive processes: a mental calculation study. *Neurosci Lett, 483,* 11-15.

[2] Laskaris NA and Ioannides AA. (2001). Exploratory data analysis of evoked response single trials based on minimal spanning tree. *Clin. Neurophysiol. 112,* 698-712.

[3] Leuchter AF, Cook IA, Newton TF, Dunkin J, Walter DO, Rosenberg-Thompson S, Lachenbruch PA, and Weiner H (1993). Regional differences in brain electrical activity in dementia: use of spectral power and ratio measures *Electroencephalogr. Clin. Neurophysiol. 87,* 385–93

[4] Ragwitz, M and H. Kantz, H (2002). Markov models from data by simple nonlinear time series predictors in delay embedding spaces. *Phys. Rev. E,* 65, 056201.

[5] Richman, JS and Moorman, JR (2000). Physiological time-series analysis using approximate entropy and sample entropy. *Am. J. Physiol: Heart & Circul. Physiol. 278,* H2039–49.

[6] Rodriguez G, Copello F, Vitali P, Perego G and Nobili F (1999). EEG spectral profile to stage Alzheimer's disease. *Clin. Neurophysiol. 110,* 1831–7

[7] Scheeringa, R., Bastiaansen, M.C.M., Petersson, K.M., Oostenveld, R., Norris, D.G., and Hagoort P. (2008). Frontal theta EEG activity correlates negatively with the default mode network in resting state. *Int. J. Psychophysiol. 67,* 242–251

[8] Nolte G, Bai O, Wheaton L, Mari Z, Vorbach S, and Hallett M. (2004). Identifying true brain interaction from EEG data using the imaginary part of coherency. *Clin Neurophysiol. 115,* 2292–2307.
